# A Novel Metal-Based Imaging Probe for Targeted Dual-Modality SPECT/MR Imaging of Angiogenesis

**DOI:** 10.3389/fchem.2018.00224

**Published:** 2018-06-20

**Authors:** Charalampos Tsoukalas, Dimitrios Psimadas, George A. Kastis, Vassilis Koutoulidis, Adrian L. Harris, Maria Paravatou-Petsotas, Maria Karageorgou, Lars R. Furenlid, Lia A. Moulopoulos, Dimosthenis Stamopoulos, Penelope Bouziotis

**Affiliations:** ^1^Radiochemical Studies Laboratory, Institute of Nuclear & Radiological Sciences & Technology, Energy & Safety, National Center for Scientific Research “Demokritos,”, Athens, Greece; ^2^Research Center of Mathematics, Academy of Athens, Athens, Greece; ^3^First Department of Radiology, School of Medicine, National and Kapodistrian University of Athens, Athens, Greece; ^4^Weatherall Institute of Molecular Medicine, University of Oxford, Oxford, United Kingdom; ^5^Department of Solid State Physics, National and Kapodistrian University of Athens, Athens, Greece; ^6^Department of Medical Imaging, Center for Gamma-Ray Imaging, University of Arizona, Tucson, AZ, United States; ^7^College of Optical Sciences, University of Arizona, Tucson, AZ, United States; ^8^Institute of Nanoscience and Nanotechnology, National Center for Scientific Research “Demokritos,” Athens, Greece

**Keywords:** SPECT/MRI, bevacizumab, iron oxide nanoparticles, angiogenesis, dual-modality imaging

## Abstract

Superparamagnetic iron oxide nanoparticles with well-integrated multimodality imaging properties have generated increasing research interest in the past decade, especially when it comes to the targeted imaging of tumors. Bevacizumab (BCZM) on the other hand is a well-known and widely applied monoclonal antibody recognizing VEGF-A, which is overexpressed in angiogenesis. The aim of this proof-of-concept study was to develop a dual-modality nanoplatform for *in vivo* targeted single photon computed emission tomography (SPECT) and magnetic resonance imaging (MRI) of tumor vascularization. Iron oxide nanoparticles (IONPs) have been coated with dimercaptosuccinic acid (DMSA), for consequent functionalization with the monoclonal antibody BCZM radiolabeled with ^99m^Tc, via well-developed surface engineering. The IONPs were characterized based on their size distribution, hydrodynamic diameter and magnetic properties. *In vitro* cytotoxicity studies showed that our nanoconstruct does not cause toxic effects in normal and cancer cells. Fe_3_O_4_-DMSA-SMCC-BCZM-^99m^Tc were successfully prepared at high radiochemical purity (>92%) and their stability in human serum and in PBS were demonstrated. *In vitro* cell binding studies showed the ability of the Fe_3_O_4_-DMSA-SMCC-BCZM-^99m^Tc to bind to the VEGF-165 isoform overexpressed on M-165 tumor cells. The *ex vivo* biodistribution studies in M165 tumor-bearing SCID mice showed high uptake in liver, spleen, kidney and lungs. The Fe_3_O_4_-DMSA-SMCC-BCZM-^99m^Tc demonstrated quick tumor accumulation starting at 8.9 ± 1.88%ID/g at 2 h p.i., slightly increasing at 4 h p.i. (16.21 ± 2.56%ID/g) and then decreasing at 24 h p.i. (6.01 ± 1.69%ID/g). The tumor-to-blood ratio reached a maximum at 24 h p.i. (~7), which is also the case for the tumor-to-muscle ratio (~18). Initial pilot imaging studies on an experimental gamma-camera and a clinical MR camera prove our hypothesis and demonstrate the potential of Fe_3_O_4_-DMSA-SMCC-BCZM-^99m^Tc for targeted dual-modality imaging. Our findings indicate that Fe_3_O_4_-DMSA-SMCC-BCZM-^99m^Tc IONPs could serve as an important diagnostic tool for biomedical imaging as well as a promising candidate for future theranostic applications in cancer.

## Introduction

Through the last two decades researchers have introduced many innovations in the area of imaging and therapy. Nanoparticles (NPs) with or without conjugated target moieties have proven invaluable tools in cancer research for multimodal imaging and targeted drug delivery as well as hyperthermia and/or controlled release treatment (Lee and Chen, 2009; Bouziotis et al., 2013). More specifically, magnetite NPs (IONPs) such as iron oxide NPs have many advantages such as their controlled size, their facile chemical modification and their ability to be manipulated by an external magnetic field, resulting in a specific, topical accumulation of IONPs and a consequent T2-enhanced MRI signal. Development of such novel imaging tools bearing optimized imaging characteristics can lead to early diagnosis and thus improved patient management.

Nanotechnology-based drug delivery platforms have emerged as a “complete system” for cancer diagnosis and therapy. More specifically, NP-based drug delivery systems can improve solubility and circulation time of therapeutic agents, resulting in overcoming limitations e.g., poor pharmacokinetics, relative to conventional drug formulations (van Vlerken and Amiji, 2006; Anselmo and Mitragotri, 2016). The enhanced permeability and retention (EPR) effect is of great importance, because it can “drive” NP systems to accumulate in the disease site by extravasation through leaky blood vessels (Torchilin, 2014). However, this passive diffusion is not enough for selective nanoparticle uptake in cancer cells (Bertrand et al., 2014).

The ability of nanoparticles to effectively travel across the tumor vessel wall to the interstitial space depends on the ratio of particle size to the size of the openings (Chauhan et al., 2012). On one hand, if this ratio is small, the transport of the particle through the pores of the wall is unhindered. On the other hand, if the size of the nanoparticle is comparable to the size of the openings, the particle will interact with the pores and the transport might be severely hindered. Moreover, large nanoparticles (>40 nm in diameter) even if they manage to cross the vessel wall, they might not be able to penetrate the dense extracellular matrix of the tumor interstitial space (Pluen et al., 2001). Indeed in many tumors, a desmoplastic response might occur that can lead to excessive production of extracellular fibers resulting in limited penetration of nanoparticles (Chauhan et al., 2011).

Following another approach, and in order to overcome these problems, NPs have been conjugated/complexed with various targeting moieties (e.g., small molecules, antibodies, peptides, and aptamers) leading to increased retention and accumulation in the tumor vasculature as well as selective and efficient internalization by target tumor cells (Zhong et al., 2014). These active targeting NP platforms achieve superior specificity, providing enhanced imaging and/or therapeutic performances compared to their passive targeting analogues.

As far as diagnostic imaging is concerned, it is not feasible to obtain all the necessary information for a particular system from a single imaging modality (De Rosales et al., 2011; Lee et al., 2014). The radionuclide-based SPECT (single photon emission computed tomography) or PET (positron emission tomography) imaging techniques offer high sensitivity but poor resolution, while MRI offers high-resolution anatomical information, but suffers from low sensitivity. Using these combinations, imaging scanners nowadays are capable of providing molecular and physiological information with remarkable sensitivity using PET and SPECT radiotracers and, at the same time, anatomical information of the tissues of interest. A hybrid SPECT/MRI probe: a) has the potential to provide better probe concentration quantification from the acquired SPECT signal, and higher spatial resolution from MRI contrast, thus leading to images that offer more relevant biological information than could be acquired from either imaging modality alone (Hoffman et al., 2014) and can highlight the value of validating different imaging methods against one another. Thus, the combination of SPECT/MRI or PET/MRI can offer synergistic advantages and lead to the more accurate *in vivo* interpretation of disease and abnormalities.

Dual modality contrast agents have already started making their mark in medical imaging. The combination of PET/SPECT with MRI can offer synergistic advantages for sensitive, high-resolution, and quantitative imaging, which can lead to the more accurate, noninvasive interpretation of disease and abnormalities and early detection of various diseases e.g., cancer (Ai et al., 2016).

Simultaneous optical and MR imaging of cancers was recently investigated by labeling recombinant humanized monoclonal antibodies or high-affinity small peptides against tumor receptors, which serve as targeting ligands, with near infrared dyes, and conjugating them to MNPs for simultaneous optical and MR imaging of cancers (Lin et al., 2018). Additionally, peptide-modified gadolinium oxide nanoprobes containing fluorescein for targeted MR/optical dual-modality imaging of various cancers have been engineered (Cui et al., 2017). Live-cell imaging studies suggest that amphiphilic dual-modality nanoprobes, containing a fluorophore for optical imaging and a metal ion chelator complexed with Gd for MRI, can self-assemble into supramolecular nanostructures and effectively label cells (Liu et al., 2015). The formation of new blood vessels *de novo* (vasculogenesis) normally occurs in fetus and in uterus assuring the supply of nutrients and oxygen to proliferating tissues (Carmeliet, 2005). However, the formation of new blood vessels from pre-existing ones (angiogenesis) is also critical in the development of various disorders including cancer, wound healing and inflammation (Eliceiri and Cheresh, 1999). As far as carcinogenesis is concerned many molecules and receptors are involved in angiogenesis regulation providing important targets for tumor diagnosis and therapy. Vascular endothelial growth factor (VEGF) is a signal protein that stimulates angiogenesis, which can contribute to tumorigenesis when it is overexpressed. The VEGF family of glycoproteins comprises five members, with the VEGF-A being the essential one for the growth and metastasis of tumors. The recombinant humanized monoclonal antibody Bevacizumab (BCZM) is an angiogenesis inhibitor that blocks angiogenesis by binding to VEGF-A (Los et al., 2007). It was developed from a murine antibody (A 4.6.1) and humanized, while retaining the specificity of the original molecule.

BCZM has been approved for the treatment of a variety of metastatic cancers and can be an ideal molecule that can target tumor sites by VEGF-A targeting (Shih and Lindley, 2006). Even though a lot of research has been performed on *in vivo* NP distribution, only a few studies are available on antibody-targeted NPs (Schroeder et al., 2012; Karmani et al., 2013). In the present study we investigated the efficacy of conjugating BCZM onto IONPs and the radiolabeling of the functionalized nanosystems with ^99m^Tc, for imaging VEGF-expressing tumors. The radiolabeled nanoformulations were evaluated *in vitro* as well as *in vivo* in tumor angiogenesis models. Active targeting afforded by Fe_3_O_4_-DMSA-SMCC-BCZM-^99m^Tc was evaluated in M165 tumor-bearing mice, in comparison to the non-specific Fe_3_O_4_-DMSA-^99m^Tc IONPs. In order to prove the efficacy of the targeted approach, preliminary imaging studies on tumor-bearing animals using MRI and gamma-ray scintigraphy were performed, which demonstrate the potential of Fe_3_O_4_-DMSA-SMCC-BCZM-^99m^Tc for targeted dual-modality imaging.

## Materials and methods

### Chemicals

All chemicals were reagent grade and were used as such unless otherwise noted. Iron(II) chloride tetrahydrate (FeCl_2_.4H_2_O, Reagent Plus, 99%) and iron(III)chloride (FeCl_3_, reagent grade, 97%) were purchased from Sigma-Aldrich. Analytical grade NH_4_OH was purchased from AnalytiCals (Carlo Erba). Purified deionized water was prepared by the Milli-Q system (Millipore Co., Billerica, MA, USA). Technetium-99m, in the form of Na^99m^TcO_4_ in physiological saline, was eluted from a commercial ^99^Mo/^99m^Tc generator acquired from GE Healthcare Ltd (United Kingdom). The monoclonal antibody Bevacizumab (Avastin®) was purchased from Roche (United Kingdom. Sulfo-SMCC (sulfosuccinimidyl 4-(N-maleimidomethyl)cyclohexane-1-carboxylate) was purchased from Thermo Fischer Scientific, while *meso*-2,3-Dimercaptosuccinic acid and human serum were acquired from Sigma-Aldrich (St. Louis, MO, USA). The human embryonic kidney cell line HEK293 was acquired from the American Type Culture Corporation, while the MDA MB 231 breast cancer cells transfected with the VEGF-165 isoform (M165) were donated by Cancer Research UK. For cell culturing, Dulbecco's modified Eagle medium (DMEM), fetal bovine serum (FBS), penicillin/streptomycin, L-glutamine, and trypsin/EDTA solution were purchased from PAA Laboratories (GmbH, Austria). The MTT tetrazolium salt, (3-[4,5-dimethylthiazol-2-yl)-2,5-diphenyltetrazolium bromide, was acquired from Thermo Fisher Scientific (Cat. No. M6494).

### Equipment

Radioactivity of the ${\text{TcO}}_{4}^{-}$ eluent was measured using a dose calibrator (Capintec, Ramsey, NJ). Thin-layer chromatography (TLC) silica gel 60 sheets (5 × 10 cm) were purchased from Merck (Darmstadt, Germany) and along with a Radio-TLC Scanner (Scan-Ram, LabLogic, Sheffield, UK) were used in the determination of radiolabeling yield/purity and *in vitro* stability studies. Radiolabeling yield of the radiolabeled antibody was assessed by HPLC (Waters). PD-10 columns (GE Healthcare), containing Sephadex G-25 resin, were used for the purification of the mercaptoethanol-reduced monoclonal antibody, while Amicon filters (cut-off value: 100 kD) were used for purification of the radiolabeled antibody. Water was deionized to 18 MΩ·cm using an easy-pure water filtration system (Barnstead International, Dubuque, Iowa). A gamma scintillation counter, a Packard Cobra II, was used to measure the radioactivity of each organ and blood samples in *ex vivo* biodistribution studies. An AXIOS-150/EX (Triton Hellas) dynamic light scattering (DLS) apparatus equipped with a 30 mW He-Ne laser emitting at 658 nm and an Avalanche photodiode detector at an angle of 90° was used for the determination of the size distributions of the nanoparticles.

Planar Scintigraphy studies were performed on a compact, high-resolution, gamma-ray camera developed at the Center for Gamma-Ray Imaging of the University of Arizona. Details of the system can be found elsewhere (Furenlid et al., 2004, 2005; Chen et al., 2005). The system comprises a 5-mm thick NaI(Ti) scintillation crystal, a 12-mm thick quartz light guide, a 3 × 3 array of 1.5-inch diameter photomultiplier tubes (PMTs), and a 40-mm thick lead parallel-hole collimator with hexagonal holes 1 mm in diameter. The system achieves a spatial resolution of about 2.5 mm at the collimator. The field-of-view of the camera is 4.0 × 4.0 in, enough to image a whole mouse without any translation.

MRI studies were performed on a 3 Tesla MRI Unit (Philips Ingenia, Philips Medical Systems, Best, The Netherlands).

### Experimental

#### Synthesis of Fe_3_O_4_ IONPs

Superparamagnetic iron oxide nanoparticles (IONPs) were synthesized in-house, according to a previously reported procedure by mixing 40.5 mg (0.25 mmol) anhydrous FeCl_3_ (MW 160.20) and 49.7 mg (0.25 mmol) FeCl_2_.4H_2_O) (MW 198.81) in ultrapure water (Stamopoulos et al., 2007). Subsequently, the complete precipitation of the IONPs was achieved by the abrupt addition of 1.5 ml of a solution of NH_4_OH (NH_4_OH:H_2_O 1:2) to the suspension (pH 9-9.5). The vials were immediately sealed, to avoid exposure to atmospheric O_2_ and were intensely stirred. The solution was subjected to magnetic decantation followed by repeated washing with distilled water (pH 6.5).

#### Functionalization of IONPs with DMSA

Fe_3_O_4_ IONPs (1.0 mL, 0.008 mmol) were incubated with 0.027 mmol DMSA dissolved in 1 mL DMSO and left overnight on a stirring apparatus at RT. The supernatant was then removed by magnetic retraction of the NP-DMSA, the nanoconjugate was washed thrice with deionized water and then reconstituted in 1 mL ultrapure water.

#### Conjugation of BCZM to NP-DMSA

Sulfosuccinimidyl-4-(N-maleimidomethyl)cyclohexane-1-carboxylate (Sulfo-SMCC) was used as the crosslinker between the NP-DMSA and BCZM. For conjugation of the antibody with the crosslinker, 50 μL of sulfo-SMCC (4.8 mg/mL in H_2_O) were added to an aliquot containing ~500 μg (0.003 μmol in 1 mL PBS) BCZM. The reaction mixture was incubated for 30 min at RT. The excess crosslinker was removed by centrifugation, using an Amicon filter with a 100 kD cut-off value. The purified SMCC-BCZM was then added to 1 mL NP-DMSA and incubated for 30 min at RT, under mechanical stirring. The supernatant was then removed by magnetic retraction of the NP-DMSA-SMCC-BCZM, the nanoconjugate was washed thrice with deionized water and then reconstituted in 1 mL deionized water, for further use in characterization and cytotoxicity experiments.

#### Size analysis of Fe_3_O_4_, Fe_3_O_4_-DMSA and Fe_3_O_4_-DMSA-SMCC-BCZM IONPs

Dynamic light scattering (DLS) was used to measure the size distributions of Fe_3_O_4_, Fe_3_O_4_-DMSA and Fe_3_O_4_-DMSA-SMCC-BCZM. nanoparticles in aqueous solutions using a DLS apparatus.

In a typical DLS measurement, 100 μL of Fe_3_O_4_ IONPs (1.25 mg/ml) diluted with 400 μL ultrapure water were measured at 22°C. Furthermore, 2 mg of Fe_3_O_4_-DMSA IONPs diluted with 800 μL ultrapure water and 0.1 mg of Fe_3_O_4_-DMSA-SMCC-BCZM NPs diluted with 400 μL ultrapure water were also measured at 22°C. For each dispersion, at least 10 light scattering measurements were collected and the results were averaged.

#### Magnetic properties of Fe_3_O_4_, Fe_3_O_4_-DMSA and Fe_3_O_4_-DMSA-SMCC-BCZM IONPs

Magnetic measurements of Fe_3_O_4_, Fe_3_O_4_-DMSA and Fe_3_O_4_-DMSA-SMCC-BCZM powder samples were carried out by means of a SQUID magnetometer (5.5 T MPMS, Quantum Design). Magnetization vs. magnetic field, M(H) was performed both at *T* = 300 K and *T* = 10 K, while magnetization vs. temperature, M(T), was measured at *H* = 50 Oe in both the zero-field cooling (ZFC) and field cooling (FC) modes (ZFC: cooling the sample to the desired temperature under zero magnetic field and then starting measuring its magnetization while progressively increasing the temperature; FC: cooling the sample to the desired temperature under the presence of a specified magnetic field while simultaneously measuring its magnetization).

#### Antibody conjugation

UV-Vis spectrophotometry was used to evaluate the conjugation between Fe_3_O_4_-DMSA and SMCC-BCZM. All samples were prepared by addition of SMCC-BCZM to Fe_3_O_4_-DMSA, intense vortexing for 1 min and consequent shaking for 30 min at RT. To a stable concentration of Fe_3_O_4_-DMSA (3 mmol/L), different concentrations of SMCC-BCZM were added, ranging from 0.0625 to 0.75 mg/mL. BCZM concentration was measured in the supernatant, after magnetic retraction of the nanoparticles. A reference BCZM solution (*C* = 1 mg/mL) was used in the control experiment, i.e., in the absence of nanoparticles.

### *In vitro* cytotoxicity study of Fe_3_O_4_-DMSA-SMCC-BCZM IONPs

Two epithelial cell lines were used for the evaluation of cytotoxicity of Fe_3_O_4_-DMSA-SMCC-BCZM IONPs, namely HEK293T (ATCC CRL-3216) and MDA MB 231 breast cancer cells transfected with the VEGF-165 isoform (M165). HEK293 cells are used as the control group (non-cancerous cell line) in our experiments. Both cell lines were cultured in standard DMEM complete (10% FBS, 1% penicillin/streptomycin, 1% L-glutamine). Cells were incubated at 37°C in a humidified atmosphere of 5% CO_2_.

#### MTT assay

As a general protocol, 5,000 cells/well were seeded in 96-well plates (Corning-Costar, Corning, NY) and cultured overnight. The positive control of this study had cells with culture medium, which were not exposed to IONPs. The two different cell lines were treated with various concentrations of Fe_3_O_4_-DMSA-SMCC-BCZM IONPs (3, 5, 10, 20, 25, 30, 35, 40, 45, 50, 100, 200, 500, and 1,000 μg/ml) for 24 h. Subsequently, the cells were rinsed once and incubated at 37°C with 100 μl serum-free medium, containing 0.5 mg/mL MTT. After 2 h, 100 μl of SDS-HCl was added to each well, mixed with the pipette and incubated for at least 1 h at 37°C. The optical densities were read at 570 nm (reference filter was set at 690 nm), on an ELISA reader.

### Radiolabeling of Fe_3_O_4_ IONPs with technetium-99m

#### Radiolabeling of Fe_3_O_4_-DMSA with technetium-99m: passive delivery of nanoparticles

Radiolabeling of Fe_3_O_4_-DMSA with ^99m^Tc was performed *via* the precursor ^99m^Tc-gluconate, as follows: A solid mixture containing 1.0 g Na gluconate, 2.0 g NaHCO_3_ and 0.015 g SnCl_2_ was homogenized and kept dry. A quantity of 0.003 g of the above mixture was dissolved in 1.0ml of a Na^99m^TcO_4_ solution, containing 370 MBq (10 mCi) of ^99m^Tc (Varvarigou et al., 2002). An aliquot of the above solution (100 μL) containing ~37 MBq of the reduced ^99m^Tc was added to 8 μmol Fe_3_O_4_-DMSA. The mixture was left at RT and the exchange reaction was completed in 30 min. The radiolabeling yield was determined by thin layer chromatography analysis (TLC). ITLC-SG was used as the stationary phase. The strips were developed using MEK and a 3/5/1.5 mixture pyridine/acetic acid/H_2_O as the mobile phases, to determine free pertechnetate, ^99m^TcO_2_ and Fe_3_O_4_-DMSA-^99m^Tc. With MEK, pertechnetate ions migrate with the solvent front, while in pyridine/acetic acid/H_2_O ^99m^TcO_2_ remains at the spot and Fe_3_O_4_-DMSA-^99m^Tc migrates to the front. The radiolabeled sample was purified by magnetic retraction. After washing twice with deionized water, the supernatant was removed and the radiolabeled sample was redispersed in deionized water. The % radiochemical purity of Fe_3_O_4_-DMSA-^99m^Tc was determined by TLC, as previously described. A control test was also carried out, in the absence of Fe_3_O_4_-DMSA-^99m^Tc.

#### Radiolabeling of BCZM with ^99m^Tc and consequent conjugation to Fe_3_O_4_-DMSA: active targeting of VEGF-A expression

BCZM-SMCC was prepared as described above. Thereafter, in order to adequately prepare BCZM for consequent radiolabeling with ^99m^Tc, its endogenous disulfide bonds were partially reduced with 2-mercaptoethanol. Briefly, BCZM-SMCC (500 μL, C_Ab_ = 2.5 mg/mL) was incubated with 25 μL 2-mercaptoethanol (1,000:1 molar ratio) for 30 min at RT. The reduced antibody conjugate was purified by centrifugation (12,000 rpm, 10 min, AMICON filters MWCO: 100kD) and reconstituted in deionized water. Labeling of BCZM-SMCC was afforded by the addition of the ^99m^Tc-gluconate precursor (100 μL/37 MBq) (Varvarigou et al., 1996). The radiolabeling yield was determined by HPLC performed on a Waters HPLC system using a TSKgel G3000SWXL size exclusion column (TOSOH Bioscience, Germany). As the eluent, PBS pH 7.4 was used at a flow rate of 0.8 mL/min. Finally, ^99m^Tc-BCZM-SMCC was added to 8 μmol Fe_3_O_4_-DMSA, intensely vortexed for 1 min, and shaken at RT for 30 min on a mechanical shaker. The radiolabeled sample was purified by magnetic retraction. After washing thrice with deionized water, the supernatant was removed and the radiolabeled sample was redispersed in 1 mL deionized water. The % radiochemical purity of Fe_3_O_4_-DMSA-SMCC-BCZM-^99m^Tc IONPs was determined by TLC, as previously described. A control test was also carried out, in the absence of IONPs.

#### *In vitro* stability studies of Fe_3_O_4_-DMSA and Fe_3_O_4_-DMSA-SMCC-BCZM-^99m^Tc IONPs

To evaluate the *in vitro* stability of Fe_3_O_4_-DMSA-^99m^Tc and Fe_3_O_4_-DMSA-SMCC-BCZM-^99m^Tc, 10 μL samples of each of the radiolabeled nanoconjugates were incubated with 90 μL phosphate buffer saline (PBS) pH 7.4 while shaking at RT. For serum stability studies, 50 μL of each of the radiolabeled nanoconjugates were incubated with 450 μL human serum at 37°C. *In vitro* stability was determined at four time points (1, 2, 4, and 24 h) by TLC, as described above. All experiments were performed in triplicate.

#### *In vitro* cell binding assay

*In vitro* cell binding experiments were performed on M165 cells, in order to assess the targeting capability of both BCZM-functionalized Fe_3_O_4_-DMSA-SMCC-BCZM-^99m^Tc and non-functionalized Fe_3_O_4_-DMSA-^99m^Tc (Orocio-Rodríguez et al., 2015; Mendoza-Nava et al., 2016). Cells were cultured as described above for the cytotoxicity experiment. On the day prior to experiments, M165 cells were seeded in 24-well plates and grown to confluency. For the binding experiment, 50 μL (0.4 μmol) of either nanoconstruct were added to each well and incubated for 1 h at 37°C. Subsequently the supernatant was removed, the cells were washed 3 times with ice-cold PBS and lysed by the addition of 1 M NaOH. Activity was measured, along with an aliquot with the initial activity, representing 100% added activity. The percent cell uptake was then calculated. Non-specific binding was determined in parallel, in the presence of a large excess of unlabeled BCZM, with 1 h of pre-incubation.

#### *Ex vivo* biodistribution studies of bare (Fe_3_O_4_-DMSA-^99m^Tc) and antibody-functionalized (Fe_3_O_4_-DMSA-SMCC-BCZM-^99m^Tc) IONPs

Animal experiments were carried out according to European and national regulations. These studies have been further approved by the Ethics Committee of the NCSR “Demokritos” and animal care and procedures followed are in accordance with institutional guidelines and licenses issued by the Department of Agriculture and Veterinary Policies of the Prefecture of Attiki (Registration Numbers: EL 25 BIO 022 and EL 25 BIO 021). Immunodeficient SCID mice were obtained from the breeding facilities of the Institute of Biosciences and Applications, NCSR “Demokritos.” The animals were housed in air-conditioned rooms under a 12-h light/dark cycle and allowed free access to food and water.

For the development of experimental tumor models, female SCID mice of 8 weeks on the day of inoculation were subcutaneously inoculated with M165 cells (1 × 10^7^ cells). Approximately 2 weeks after inoculation, *ex vivo* biodistribution studies and *in vivo* imaging studies on the tumor-bearing mice were performed. Fe_3_O_4_-DMSA-^99m^Tc and Fe_3_O_4_-DMSA-SMCC-BCZM-^99m^Tc were evaluated in 2 groups of tumor-bearing SCID mice (*n* = 4 mice, animal weight 18–20 g). The nanoradiotracers were intravenously administered via the tail vein. Each mouse received 100 μL (0.8 μmol/89 μg/2.0 ± 0.3 MBq) of either Fe_3_O_4_-DMSA-^99m^Tc (passive delivery) or Fe_3_O_4_-DMSA-SMCC-BCZM-^99m^Tc (active targeting). The animals were euthanized at 2, 4, and 24 h post-injection (4 mice per time-point) and the organs of interest, as well as the tumor, were removed, weighed and counted in a NaI well-counter, along with samples of blood, muscles and urine. The remaining radioactivity in the tail, as well as background counts were subtracted, and the radioactivity decay was auto-corrected by the counter. The accumulation of radiolabeled MNPs in each organ was expressed as the percentage injected dose per gram of tissue (%ID/g ± SD) and calculated compared to the activities of a standard dose of the injected solution.

Binding specificity of Fe_3_O_4_-DMSA-SMCC-BCZM-^99m^Tc (the targeted nanoconstruct) was assessed in M165 tumor-bearing mice (*n* = 4 mice), after pre-injection of the mice with 2.5 mg (0.016 μmol)/100 μL “cold” bevacizumab 24 h before injection of the radiotracer. These animals were euthanized at 4 h post-injection of Fe_3_O_4_-DMSA-SMCC-BCZM-^99m^Tc and were assessed as described above.

#### *In vivo* imaging studies (pilot studies) gamma camera

All animals were bearing a tumor in their left front limb. After tracer injection, the animals were anesthetized with an intraperitoneal (IP) injection of ketamine (75 mg/kg) and xylazine (5 mg/kg) and placed on the prone position on the camera face. Planar scintigraphy was performed by collecting a 60-min image following tracer injection. Furthermore, all four animals were imaged 24 h after injection by acquiring a 60-min image.

#### MR imaging

MRI exams were conducted at the First Department of Radiology of the National and Kapodistrian University of Athens on a 3 Tesla MRI unit, using a surface phased-array coil.

MRI experiments were performed on 3 groups of M165 tumor-bearing SCID mice, as follows: Group 1: Mice injected with Fe_3_O_4_-DMSA-SMCC-BCZM (1 mg IONPs/100 μL, *n* = 3 mice); Group 2: Mice injected with Fe_3_O_4_-DMSA (1 mg IONPs/100 μL *n* = 3 mice); and Group 3: Mice injected with saline (*n* = 2 mice). Anesthesia was induced with a mixture of ketamine/xylazine. T2-Weighted images were acquired before the injection of nanoparticles. The next day mice were injected with Fe_3_O_4_-DMSA-SMCC and Fe_3_O_4_-DMSA-SMCC-BCZM and were imaged 4 h post-injection with the same parameters. MR images were reviewed and signal intensity measurements were performed on a dedicated workstation using diagnostic software (Philips Intellispace Portal v9, Philips Healthcare, Best, The Netherlands).

#### Histopathology studies

Twenty-four hours post-MRI scanning, the mice were euthanized and the tumors, livers, lungs and kidneys of each mouse were surgically removed. The tissues were then fixed in 10% formalin, embedded in paraffin, sectioned at 6 μm and stained with Perl's reagent (Prussian Blue). The slides and the pictures of each tissue were taken using an Olympus U-TVO.5XC-3 microscope, equipped with an Infinity1 Lumen*era* camera (magnification × 40).

### Statistical analysis

The data are presented as means ± standard deviations. For the biodistribution studies, the data were compared using an unpaired *t*-test with a significance level of *P* < 0.05. All analyses were performed using Microsoft Office Excel.

## Results and discussion

The aim of the present study was to show that bevacizumab-functionalized IONPs show specific accumulation in VEGF-A expressing tumors, while their non-functionalized counterparts exhibit limited uptake due to the EPR effect. The primary idea behind the design and synthesis of these NPs was to provide a platform for the development of a dual-modality molecular imaging agent.

### Synthesis and characterization of Fe_3_O_4_ IONPs

**Figure 1:**
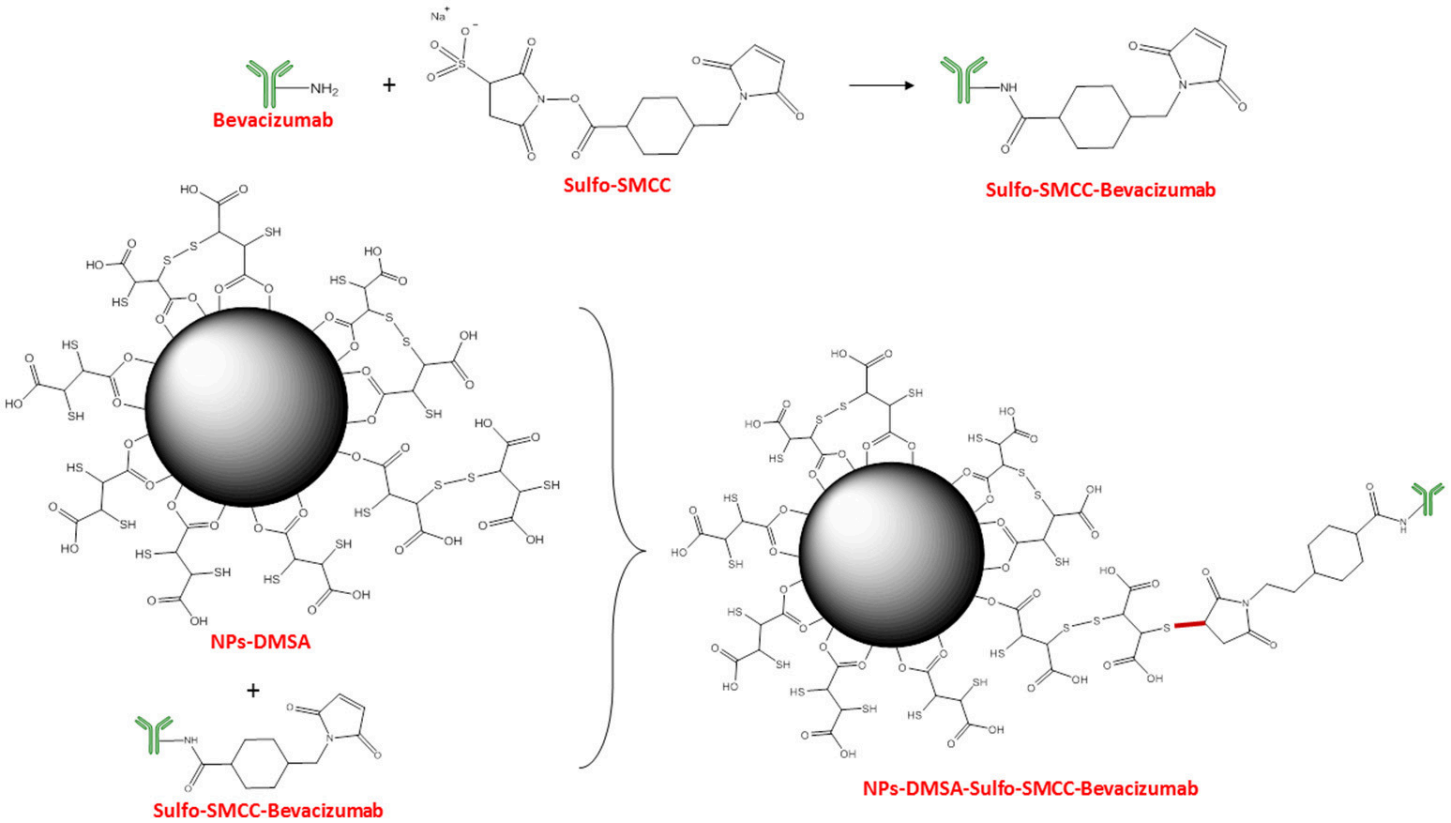
Schematic representation of Fe_3_O_4_-DMSA-SMCC-BCZM nanoparticles (FNs).

The main reason for coating the nanoparticle surface with various molecules is to render them non-toxic, biocompatible and stable (Barrow et al., 2015). 2,3-dimercaptosuccinic acid (DMSA) has been repeatedly used for surface-functionalizing inorganic NPs, as an effective way to increase their biocompatibility (Fauconnier et al., 1997; Auffan et al., 2006; Wang et al., 2011). DMSA is an FDA-approved, orally administered metal-chelating agent often used to coat iron oxide NPs, in order to improve their stability and biocompatibility (Chen et al., 2008). Apart from providing nanoparticle stability under physiological conditions, DMSA offers two functional groups, namely –COOH and –SH, which can both be exploited for the covalent bonding of a variety of organic molecules (Ruiz et al., 2014; Galli et al., 2017). In this study, IONPs were synthesized according to the co-precipitation method, while their surface was modified with DMSA for further functionalization with BCZM-SMCC (Figure 1). The ligand DMSA binds to the nanoparticle surface in a bidentate manner through oxygen atoms, and the created Fe-O-C bond is similar to a polar covalent bond (Figure 1; Ruiz et al., 2014). Conjugation of BCZM to the thus formed NP-DMSA was accomplished *via* the crosslinker Sulfo-SMCC. Sulfo-SMCC contains an amine-reactive N-hydroxysuccinimide (NHS ester) and a sulfhydryl-reactive maleimide group. The NHS esters of Sulfo-SMCC react with the primary amines of BCZM to form stable amide bonds while the maleimides react with the sulfhydryl groups of NP-DMSA to form stable thioether bonds.

### Size analysis of Fe_3_O_4_, Fe_3_O_4_-DMSA and Fe_3_O_4_-DMSA-SMCC-BCZM IONPs

**Figure 2:**
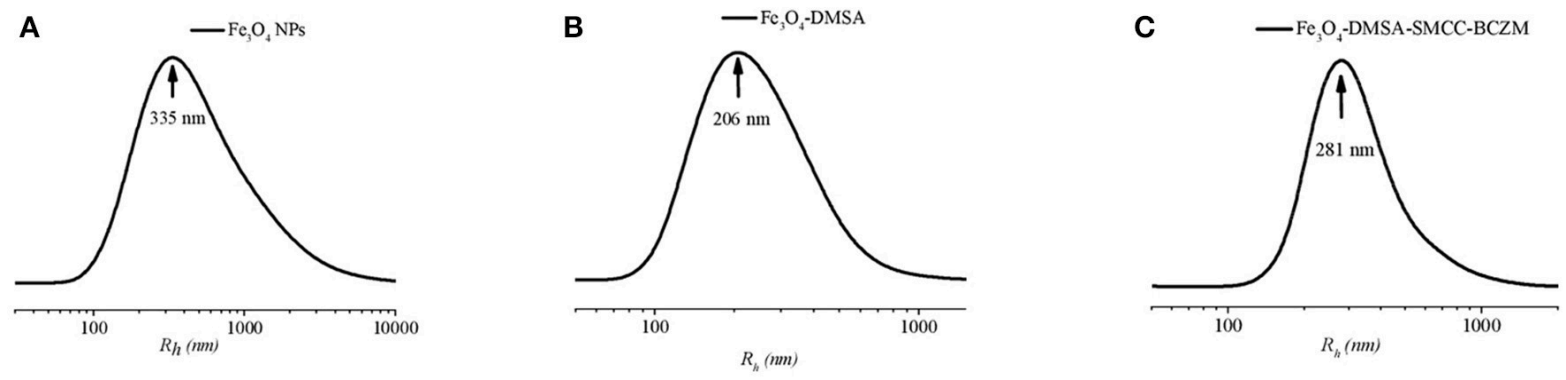
Intensity weighted hydrodynamic radii size distribution of **(A)** Fe_3_O_4_, **(B)** Fe_3_O_4_-DMSA, and **(C)** Fe_3_O_4_-DMSA-SMCC-BCZM.

DLS measurements were carried out on the Fe_3_O_4_, Fe_3_O_4_-DMSA and Fe_3_O_4_-DMSA-SMCC-BCZM IONPs (Figure 2). The samples were diluted with ultrapure water and measured at 22°C. Using CONTIN analysis of the DLS measurements, the mean size distribution of intensity weighted hydrodynamic radii of Fe_3_O_4_, Fe_3_O_4_-DMSA, and Fe_3_O_4_-DMSA-SMCC-BCZM IONPs were found to be 335, 206, and 281 nm respectively. This result indicates the general effect of surface coating, which prevents the *in vivo* aggregation of the nanoparticles as it provides sufficient electrostatic (and/or steric) repulsion between them and thus, maintains the nanoparticles apart from one another against attractive forces. The hydrodynamic size of Fe_3_O_4_-DMSA and Fe_3_O_4_-DMSA-SMCC-BCZM IONPs is close to the upper size limit (400–500 nm) for the extravasation of nanoparticles into tumor tissues, *via* the EPR effect.

### Magnetic properties of Fe_3_O_4_, Fe_3_O_4_-DMSA, and Fe_3_O_4_-DMSA-SMCC-BCZM IONPs

**Figure 3:**
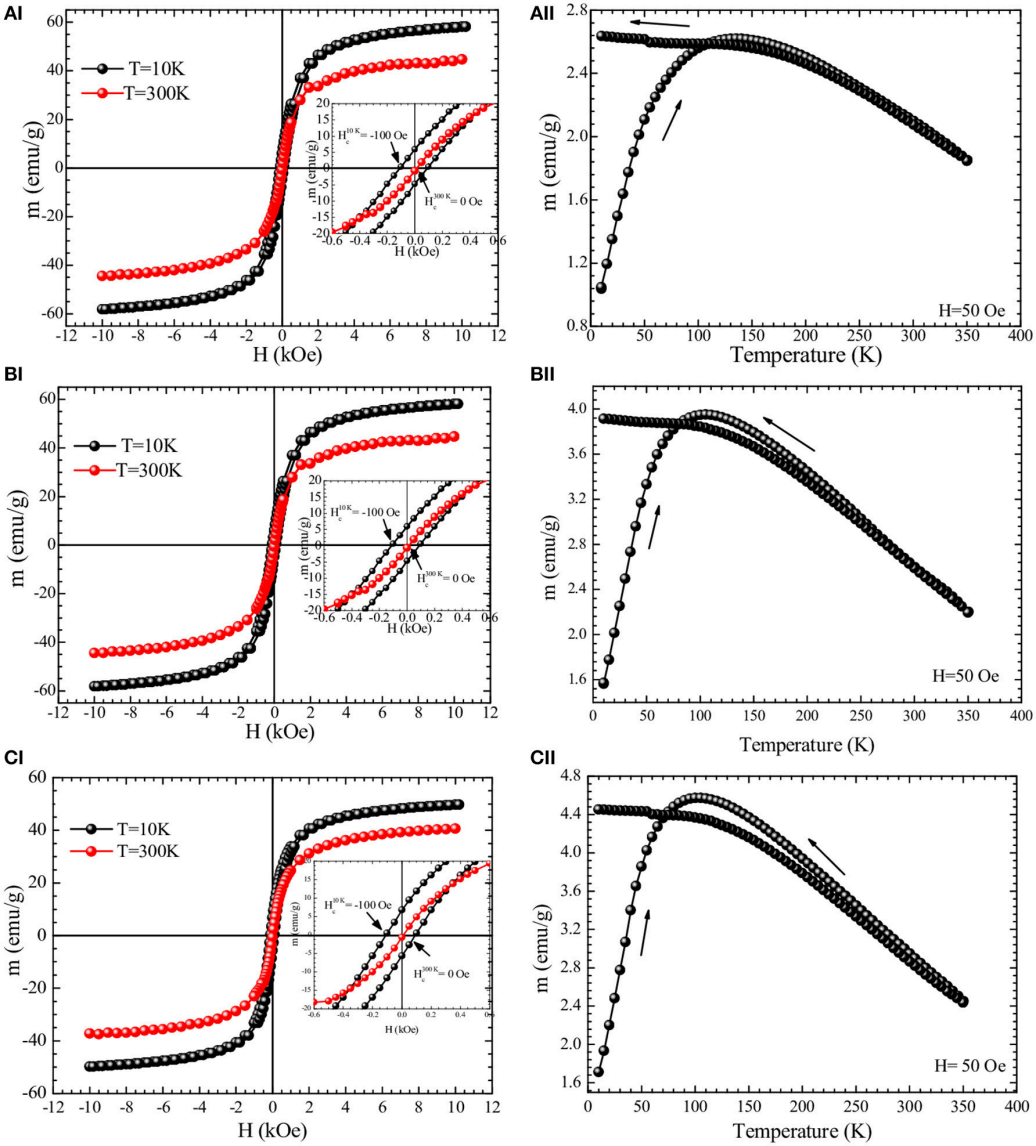
Magnetization measurements **(AI-CI)** vs. magnetic field, at 10 and 300 K, and **(AII-CII)** vs. temperature measured at *H* = 50 Oe for **(AI,AII)** Fe_3_O_4_; **(BI,BII)** Fe_3_O_4_-DMSA; and **(CI,CII)** Fe_3_O_4_-DMSA-SMCC-BCZM. All samples were in powder form.

The magnetic properties of Fe_3_O_4_, Fe_3_O_4_-DMSA, and Fe_3_O_4_-DMSA-BZCM nanoparticles, presented in Figures 3A–C respectively, were assessed by magnetization measurements vs. magnetic field, M(H), at *T* = 300 K and *T* = 10 K [panels (A.I–C.I)], as well as by ZFC-FC magnetization at 50 Oe [panels (A.II–C.II)]. The saturation magnetization, Ms, was found to be Ms = 45 emu/g, Ms = 41 emu/g and Ms = 41 emu/g at *T* = 300 K for Fe_3_O_4_, Fe_3_O_4_-DMSA, and Fe_3_O_4_-DMSA-SMCC-BZCM nanoparticles, respectively. From the comparison of the magnetization measurements, it is concluded that the magnetization of all the samples is preserved and is not affected either by the DMSA coating or by the functionalization of the nanoparticles with the antibody. The values of remanent magnetization and coercivity for all samples at 300 K are zero, which is in accordance to their superparamagnetic behavior (or more appropriately, soft ferromagnetic/ferrimagnetic character). Furthermore, the ZFC-FC magnetization curves of Fe_3_O_4_, Fe_3_O_4_-DMSA, and Fe_3_O_4_-DMSA-SMCC-BZCM nanoparticles exhibit a maximum in the ZFC curve that corresponds to the so-called blocking temperature T_B_ = 130, 100, and 100 K, respectively. These magnetization data show that the Fe_3_O_4_ IONPs preserve their magnetic properties (high saturation magnetization and zero coercivity) upon functionalization and thus are appropriate for usage in diagnostic imaging application at RT.

### Antibody conjugation

**Figure 4:**
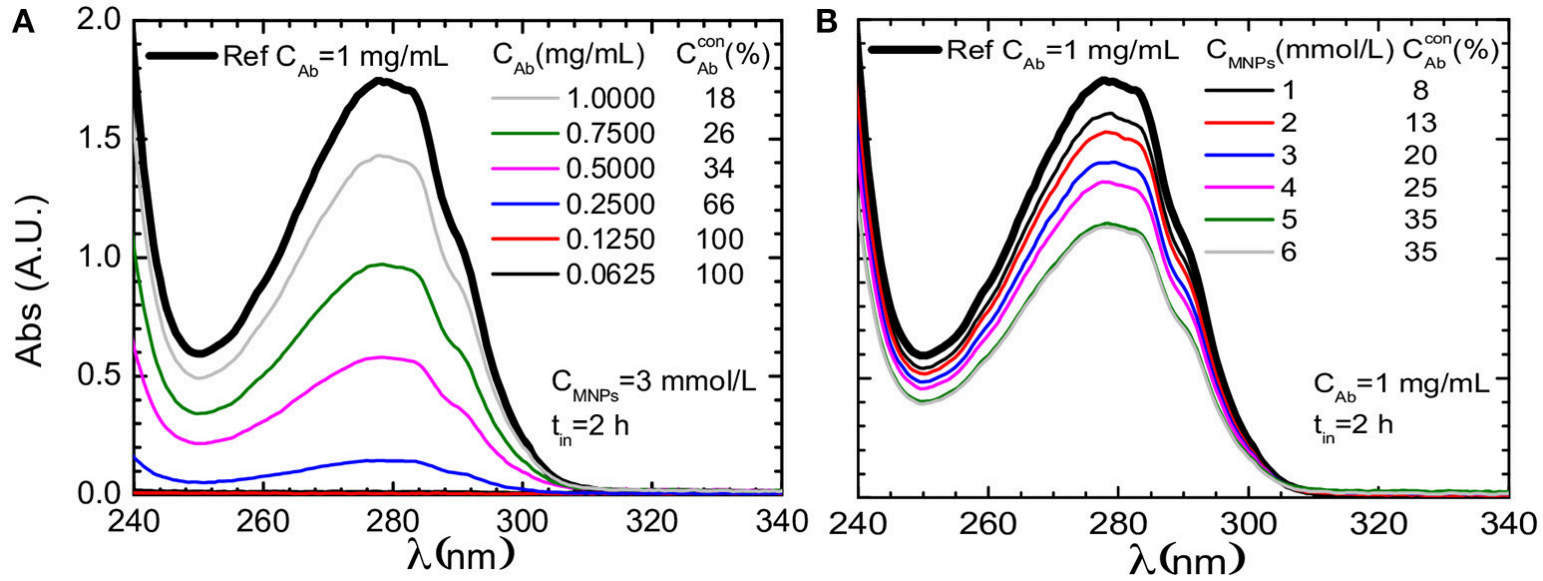
UV-vis data (focused in the range of 240–340 nm) for the supernatant of Fe_3_O_4_-DMSA-SMCC-BCZM samples that were prepared for incubation duration, time = 2 h, for six different concentrations of **(A)** BCZM and constant concentration of IONPs (C_FNs_ = 3 mmol/L) and **(B)** IONPs and constant concentration of BCZM (C_Ab_ = 3 mmol/L). The respective curve of a reference BCZM solution (1 mg/mL) is also shown, in both cases. The ${\text{C}}_{\text{Ab}}^{\text{con}}$ presented in both panels refers to the percentage of BCZM conjugated with the Fe_3_O_4_ IONPs, as this was estimated from the respective set of data. The supernatant of the samples was isolated when the magnetic species were trapped by means of an external magnetic field.

The conjugation between Fe_3_O_4_-DMSA and SMCC-BCZM may be reliably proven by means of UV-vis spectrophotometry, as previously reported by our group (Stamopoulos et al., 2007). Figures 4A,B present data for the supernatant of Fe_3_O_4_-DMSA-SMCC-BCZM samples that were prepared for incubation duration, time = 2 h. (Figure 4A) shows data for constant concentration of Fe_3_O_4_ (C_IONPs_ = 3 mmol/L) and for six different concentrations of BCZM, while (Figure 4B) shows the respective data for constant concentration of BCZM (C_Ab_ = 3 mmol/L) and six different concentrations of Fe_3_O_4_ IONPs. The data of (Figure 4A) clearly show complete conjugation of BCZM to the IONPs for antibody concentrations up to a characteristic value 0.1250 mg/mL. However, the percentage of conjugated BCZM decreases as its initial concentration increases. The data of (Figure 4B) prove that the percentage of BCZM conjugated with the IONPs increases as their concentration increases, however it reaches a plateau at a characteristic value 5 mmol/L. These data guided us to choose anoptimum concentration ratio between BCZM and IONPs, that is C_BCZM_/C_IONPs_ = 0.5 mg/mL/8 mmol/L, in our subsequent experiments.

### *In vitro* cytotoxicity study of Fe_3_O_4_-DMSA-SMCC-BCZM

**Figure 5:**
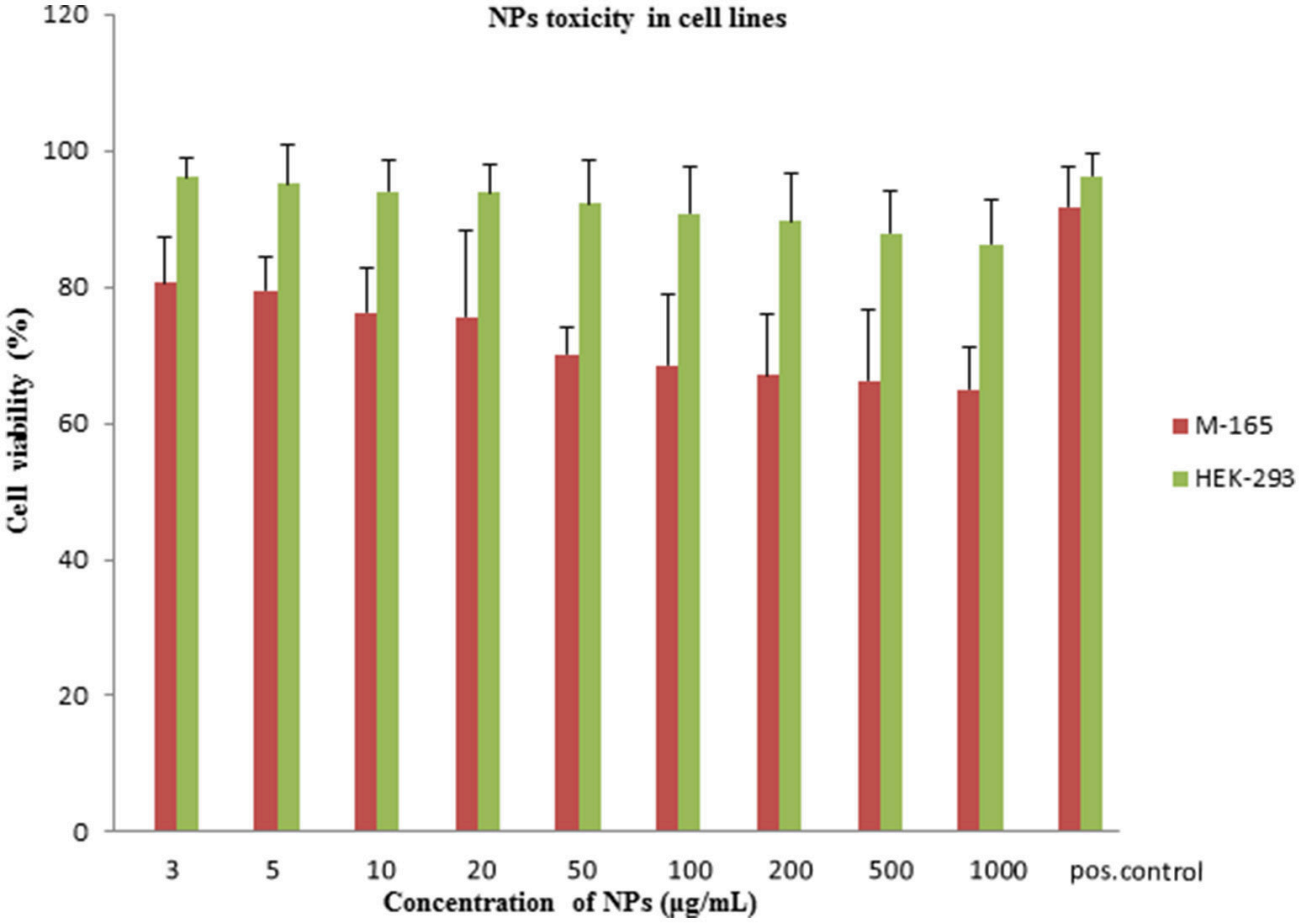
Viability assay on M165 and HEK293 cell lines. Graph of MTT assay after 24 h treatment of M-165 cells and HEK293 cells with various concentrations of NPs. Positive control shows M-165 cells and HEK293 cells without the exposure to NPs. The cell viability is expressed as % cell viability in comparison to a positive control.

Cytotoxicity of iron oxide NPs at concentrations >300 μg/mL is mainly due to the production of Reactive Oxidative Species (ROS) (Shukla et al., 2015). Quantitative assays of the metabolic activity of cancer cell lines in the presence of Fe_3_O_4_-DMSA-SMCC-BCZM could grant a better knowledge of the mechanisms implied in the toxicity caused by these IONPs. The MTT viability assay tracks the activity of reductase enzymes, thus measuring the cell viability of M165 and HEK293 cell lines, after a 24 h treatment with various concentrations of Fe_3_O_4_-DMSA-SMCC-BCZM IONPs (Jarockyte et al., 2016; Karageorgou et al., 2017). Our experiments demonstrated a similar behavior on both the M165 and HEK293 cell lines, at nanoparticle concentrations ranging from 3 to 1,000 μg/ml. Cell viability was over 65% at all concentration points for both cells lines (Figure 5). Keeping in mind that the maximum dose of administered iron oxide NPs in our study is 1,000 μg/mouse (which is the injected dose for the MR imaging study), our results suggest that our nanoconstruct does not cause toxic effects on either cell line, even at the 1,000 μg/ml concentration point. These results are in accordance with toxicity results of other DMSA-coated nanoparticles reported in the literature (Mejías et al., 2010, 2013; Ruiz et al., 2013; Paik et al., 2015; Costa et al., 2016; Shelat et al., 2018).

### Radiosynthesis and *in vitro* stability studies of Fe_3_O_4_-DMSA-^99m^Tc and Fe_3_O_4_-DMSA-SMCC-BCZM-^99m^Tc

Radiolabeling of Fe_3_O_4_-DMSA was achieved by transcomplexation of Sn^2+^-reduced [^99m^Tc]-pertechnetate in the presence of an excess of a low-affinity chelating ligand (gluconate), which ensures that ^99m^Tc binds to the exposed thiol groups. Fe_3_O_4_-DMSA-^99m^Tc was prepared at a satisfactory radiolabeling yield (70%) and high radiochemical purity (>95%) post-purification by magnetic retraction, as determined by TLC analysis (Figure S1). Labeling of the SMCC-BCZM conjugate was also performed via the ^99m^Tc-gluconate precursor at high radiochemical yield (>98%), as determined by HPLC analysis (Figure S2). Incubation of Fe_3_O_4_-DMSA with SMCC-BCZM-^99m^Tc led to the formation of Fe_3_O_4_-DMSA-SMCC-BCZM-^99m^Tc at a radiochemical purity of >92% after purification by magnetic retraction (Figure S3).

A significant factor to be considered when developing a new radiolabeled nanoparticle is that the radionuclide must be bound to the nanoparticle to form a stable conjugate under physiological conditions to avoid their separation and non-specific deposition of free ions in tissues. Otherwise, biodistribution and imaging data will not indicate the fate of nanoparticles, as the radionuclide distribution will not reflect that of the nanoparticles. With the aim to assess the *in vitro* stability of Fe_3_O_4_-DMSA-^99m^Tc and Fe_3_O_4_-DMSA-SMCC-BCZM-^99m^Tc in biological media, the radiolabeled samples were incubated with PBS and human serum. The results exhibited satisfactory *in vitro* stability in PBS (~80% stable IONPs, Figure S4) and high *in vitro* stability human serum (~90% stable IONPs, Figure S5) for both radiotracers at 24 h post-incubation, as evaluated by TLC analysis. From the *in vitro* serum stability results, it is safe to say that both radiolabeled nanoconstructs are stable enough to be used as *in vivo* imaging agents. Our results are in accordance to previously published works (De Rosales et al., 2011; Karageorgou et al., 2017).

### *In vitro* cell binding assay

The cell binding assay was performed on the M165 cell line after 24 h incubation with Fe_3_O_4_-DMSA-SMCC-BCZM-^99m^Tc and Fe_3_O_4_-DMSA-^99m^Tc (50 μL, 0.04 μmol). Targeted Fe_3_O_4_-DMSA-SMCC-BCZM-^99m^Tc had specific recognition, as cell uptake was ~4.3 times higher than for Fe_3_O_4_-DMSA-^99m^Tc, indicating the ability of the immunoconjugate to bind to the VEGF-165 isoform overexpressed on M-165 tumor cells. Specificity of Fe_3_O_4_-DMSA-SMCC-BCZM-^99m^Tc was further confirmed by blocking the cells with an excess of unlabeled BCZM (Table 1; Morales-Avila et al., 2011; Orocio-Rodríguez et al., 2015; Mendoza-Nava et al., 2016).

**Table 1:** Cell binding of Fe_3_O_4_-DMSA-SMCC-BCZM-^99m^Tc and Fe_3_O_4_-DMSA-^99m^Tc in M165 cells without (w/o) and with blockage by unlabeled BCZM, at 24 h (% of Total Activity ± SD, *n* = 3).

**Nanoradiopharmaceutical**	**Cell Uptake w/o block (%)**	**Cell uptake with block (%)**
Fe_3_O_4_-DMSA-SMCC-BCZM-^99m^Tc	25.27 ± 1.84	10.74 ± 0.88
Fe_3_O_4_-DMSA-^99m^Tc	5.88 ± 1.06	5.07 ± 1.29

In light of the performed cell binding studies, and the demonstrated specificity of Fe_3_O_4_-DMSA-SMCC-BCZM-^99m^Tc toward M165 cells overexpressing the VEGF-165 isoform, this should be the nanoconstruct of choice for further *ex vivo* and *in vivo* experimentation.

### *Ex vivo* biodistribution studies

**Figure 6:**
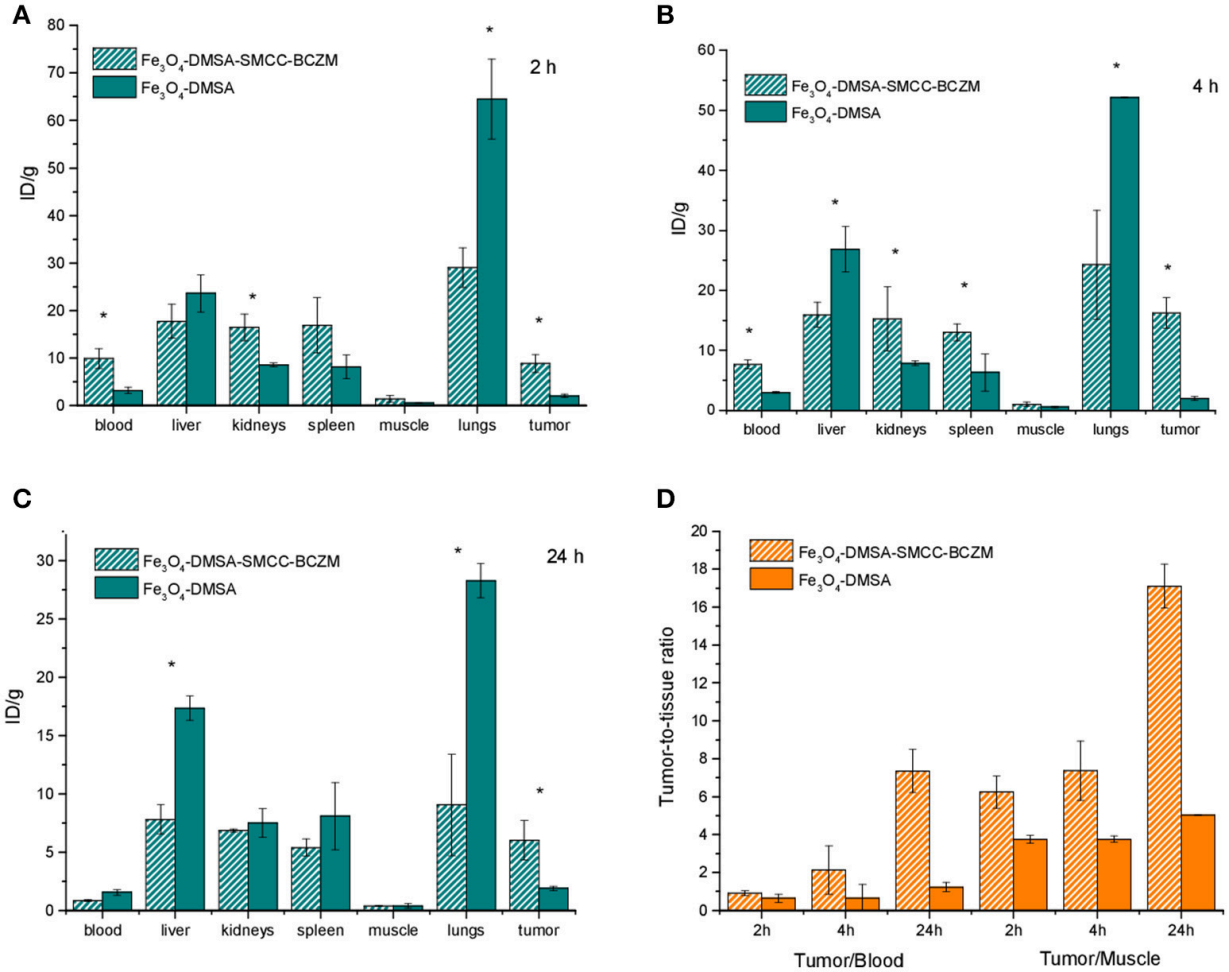
Biodistribution results of M165 tumor-bearing SCID mice injected with Fe_3_O_4_-DMSA-SMCC-BCZM-^99m^Tc and Fe_3_O_4_-DMSA-^99m^Tc at 2, 4, and 24 h p.i. **(A–C)**. Tumor-to-blood, tumor-to-liver and tumor-to-muscle ratios at 2, 4, and 24 h p.i. **(D)**. Significant differences (*P* < 0.05) are denoted with an asterisk. Numerical data provided in tabulated form in the Supplementary Materials section (Tables S1, S2).

In order to evaluate the potential of the radiolabeled NP-systems as SPECT/MRI imaging agents, *ex vivo* biodistribution experiments were performed. Following i.v. injection, the ^99m^Tc-labeled ferromagnetic NPs enter the blood stream and after circulating for some time in the body they reach a desired target i.e., tumor, depending on their surface functionalization. The degree of target uptake depends on the affinity between the target molecule and the functionalized NP-system. It is also essential that the administered nanomaterial circulates for long enough time for effective tumor uptake before being eliminated by the organism. The *ex vivo* biodistribution pattern of Fe_3_O_4_-DMSA-^99m^Tc and Fe_3_O_4_-DMSA-SMCC-BCZM-^99m^Tc was assessed in xenografted mice. Both species of IONPs were administered via tail-vein injection in M165 tumor-bearing SCID mice. The accumulation of the technetium-labeled IONPs in the organs at all time-points examined is presented in Figure 6, as percentage of injected dose per gram tissue (% ID/gr ± SD). Blood retention of the nanoparticles was 3.21 ± 0.65%ID/g and 9.90 ± 2.07%ID/g at 2h p.i. for Fe_3_O_4_-DMSA-^99m^Tc and Fe_3_O_4_-DMSA-SMCC-BCZM-^99m^Tc, respectively, while the antibody-functionalized conjugate showed a faster blood clearance at 24 h p.i. (0.81 ± 0.08%ID/g), compared to the non-functionalized IONPs (1.54 ± 0.24%ID/g). After organ distribution through blood, high levels of radioactivity were observed for both NPs in liver, spleen, kidney and lungs even, with different kinetics. In the liver, there was more pronounced uptake for Fe_3_O_4_-DMSA-^99m^Tc (23.60 ± 3.90%ID/g, 26.84 ± 3.76%ID/g and 17.33 ± 1.02% ID/g at 2, 4 and 24 h p.i.), while in the spleen the uptake was considerably lower for both radiolabeled nanoconstructs at 24 h p.i. (8.09 ± 2.88%ID/g and 5.38 ± 0.73%ID/g, for Fe_3_O_4_-DMSA-^99m^Tc and Fe_3_O_4_-DMSA-SMCC-BCZM-^99m^Tc, respectively). These findings are consistent with the observed faster blood clearance of the non-functionalized IONPs at 2 h p.i., which may be attributed to their rapid accumulation in the liver and lungs. The lower liver uptake for Fe_3_O_4_-DMSA-SMCC-BCZM-^99m^Tc at all the examined time-points (17.77 ± 3.61%ID/g, 15.88 ± 2.10%ID/g and 7.81 ± 1.25%ID/g at 2, 4 and 24 h p.i.) can be attributed to the presence of BCZM which is protecting them to a certain degree from being recognized by Kupffer cells (de Souza Albernaz et al., 2018). As concluded from the biodistribution studies, the MNP species investigated here are cleared mostly through the hepatobiliary pathway since their hydrodynamic diameter is significantly larger than the cutoff for renal filtration (~5 nm) (Hong et al., 2012). Nevertheless, there is a significant renal uptake at all time-points which can be attributed to a small extent of renal clearance. Additionally, the high radioactivity concentration in the kidneys particularly for Fe_3_O_4_-DMSA-SMCC-BCZM-^99m^Tc at 2 and 4h p.i. (16.46 ± 2.8%ID/g and 15.22 ± 5.37%ID/g respectively) can be partly attributed to the expression of VEGF-A in glomerular podocytes and in tubular epithelial cells, being essential for maintaining the glomerular filtration barrier (Turner et al., 2016). Previous work by the group of Vegt et al has shown that fragments of albumin might be suitable for inhibiting tubular reabsorption of peptides, however the effect of pre-administration of these agents on kidney uptake has not been investigated in the present study (Vegt et al., 2008). Lung uptake was high for both species at all time-points examined but it was significantly higher for the non-targeted species (64.51 ± 8.44%ID/g at 2 h p.i.), remaining quite high even at 24 h p.i. (28.27 ± 1.45%ID/g). This may be attributed to various factors, such as the size of the nanoconstructs, their DMSA coating and, for the Fe_3_O_4_-DMSA-SMCC-BCZM-^99m^Tc species, its functionalization with BCZM (Chaves et al., 2002, 2005; Monge-Fuentes et al., 2011).

In the case of Fe_3_O_4_-DMSA-SMCC-BCZM-^99m^Tc, tumor accumulation was quick, starting at 8.9 ± 1.88%ID/g at 2 h p.i., showed a slight increase at 4 h p.i. (16.21 ± 2.56%ID/g) and then decreased at 24 h p.i. (6.01 ± 1.69%ID/g). At all time-points studied, tumor uptake was significantly different (*P* < 0.05) between the two radiolabeled species, in favor of the BCZM-functionalized MPNs. Even though there is a decrease in tumor uptake in time, the tumor-to-blood ratio reached a maximum at 24 h p.i. (~7), which is also the case for the tumor-to-muscle ratio (~18) (Figure 6D). A further interesting observation is that the non-targeted Fe_3_O_4_-DMSA-^99m^Tc showed practically no decline in tumor uptake value from 4 to 24 h p.i., which leads us to the conclusion that the nanoparticle shell was stably labeled with ^99m^Tc, without being the subject of any degradation (Rainone et al., 2004). The decline in tumor uptake witnessed for Fe_3_O_4_-DMSA-SMCC-BCZM-^99m^Tc may be attributed to *in vivo* clearance of the nanoconstruct, however even if this is the case, there is significant increase in the tumor/blood ratio from 4 to 24 h p.i. Because of the high tumor contrast exhibited after functionalization of these IONPs with BCZM, showing us favorable tumor-to-background ratios, we expect to witness satisfactory imaging properties for this nanoconstruct. All other organs studied showed low or negligible uptake. The results clearly indicate that biomarker-mediated active targeting provided the best efficiency in breast-cancer-induced angiogenesis detection, compared to simple EPR passive accumulation.

Binding specificity of Fe_3_O_4_-DMSA-SMCC-BCZM-^99m^Tc was investigated by performing blocking experiments in M165 tumor-bearing mice at 4h post-injection of the radiotracer, after pre-injection of the mice with a large excess (2.5 mg) unlabeled BCZM. Tumor uptake was significantly different (*P* < 0.05) between the blocked and unblocked mice (~75% accumulation decrease in the M165 tumor), thus confirming the binding specificity of Fe_3_O_4_-DMSA-SMCC-BCZM-^99m^Tc (Figure S6, Table S3). A significant decrease in kidney uptake was also demonstrated, indicating renal VEGF-A blocking.

### *In vivo* imaging studies (pilot studies)

**Figure 7:**
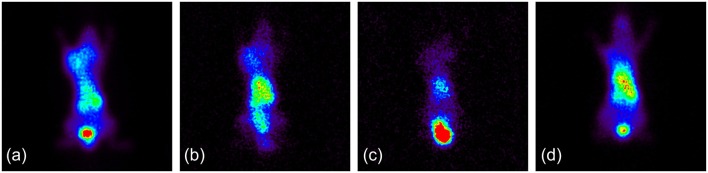
**(a)** Fe_3_*O*_4_-DMSA-SMCC-BCZM-^99m^Tc, imaging at 1 h p.i.; **(b)** Fe_3_*O*_4_-DMSA-SMCC-BCZM-^99m^Tc, imaging at 24 h p.i.; **(c)** Fe_3_*O*_4_-DMSA-SMCC-BCZM-^99m^Tc, blocking with excess BCZM 24 h prior to Fe_3_*O*_4_-DMSA-SMCC-BCZM-^99m^Tc administration, imaging at 1 h p.i.; **(d)** Fe_3_*O*_4_-DMSA-^99m^Tc, imaging at 1 h p.i.

After evidence of tumor accumulation was provided by the *ex vivo* biodistribution study, and in order to evaluate the potential of VEGF-targeting IONPs as a dual-modality imaging agent, initial gamma-ray and MR imaging were performed on M165 tumor-bearing SCID mice to verify our hypothesis. Static planar scintigraphic images of a mouse injected with Fe_3_O_4_-DMSA-SMCC-BCZM-^99m^Tc at 1 h and 24 h post injection are shown in Figures 7a,b. These pilot images show significant accumulation of the radiotracer in the tumor from the first hour p.i., which was retained at the tumor site up to 24 h p.i. However, after administration of an excess of BCZM 24 h prior to injection of Fe_3_O_4_-DMSA-SMCC-BCZM-^99m^Tc (blocking study), no tumor uptake was present 1 h p.i. (Figure 7c). Furthermore, when the animal was injected with Fe_3_O_4_-DMSA-^99m^Tc (non-targeted nanoconstruct), practically no tumor uptake was seen (Figure 7d), as expected.

**Figure 8:**
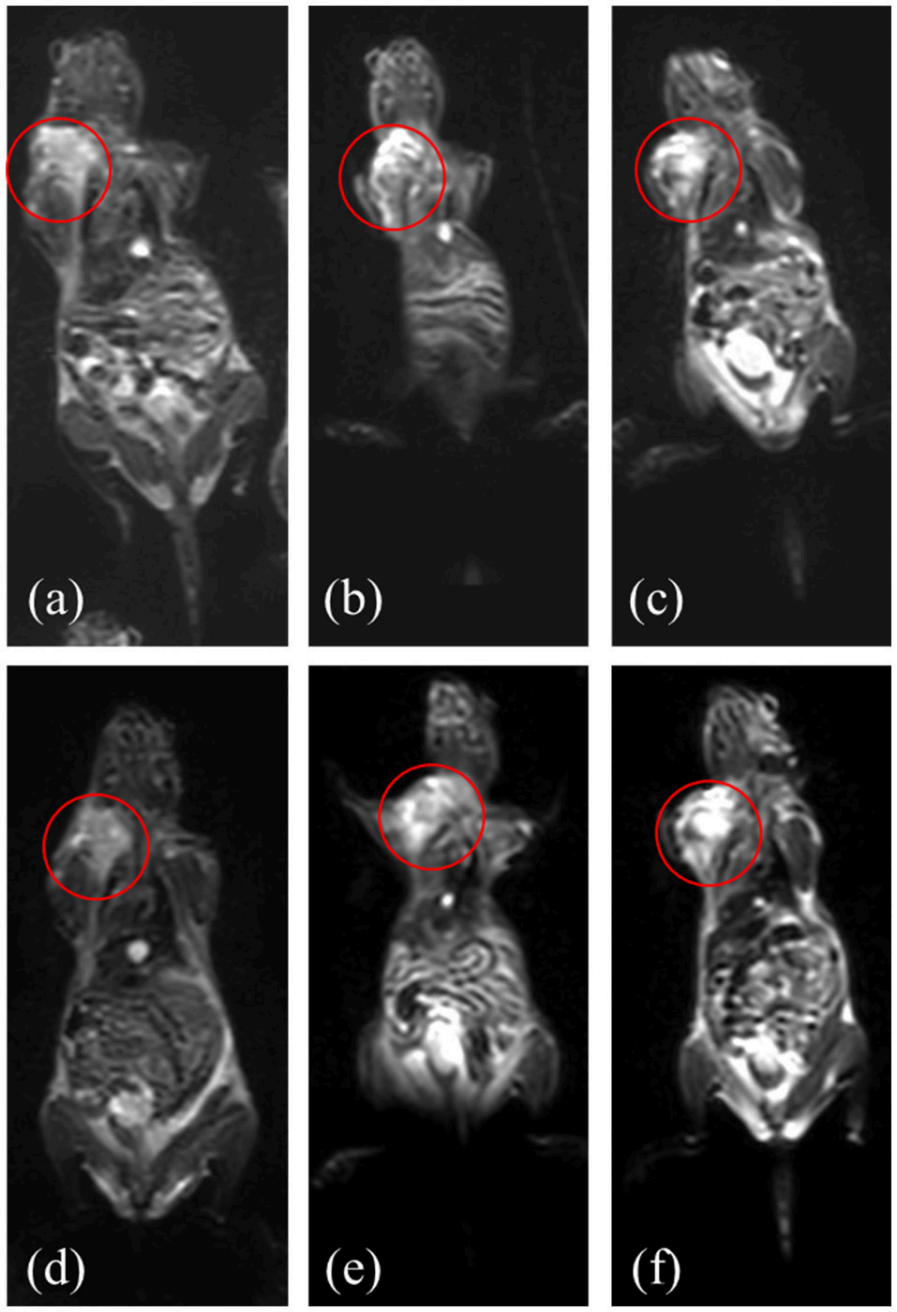
MR imaging of mice injected with Fe_3_O_4_-DMSA-SMCC-BCZM **(d)**, Fe_3_O_4_-DMSA **(e)** and without injection of nanoparticles (**f**, control mice) at 4 h p.i.; images of the mice 1 day before nanoparticle injection (**a–c**, respectively). Red circles designate the tumor area.

In the MR imaging experiment which was conducted, T2 weighted images acquired before administration of nanoparticles showed the tumors with high signal intensity. (Figures 8a–c). After nanoparticle administration the following day, decreased tumor MR signal intensity was noted, indicating specific uptake of Fe_3_O_4_-DMSA-SMCC-BCZM (Figure 8d), while the non-targeted nanoconstruct Fe_3_O_4_-DMSA showed only a slight drop in signal intensity (Figure 8). M165 tumor-bearing mice which were not injected with nanoparticles served as our control mice, and exhibited no drop in tumor signal intensity (Figure 8).

One of the most significant advantages of SPECT is its sensitivity, which is the reason why SPECT imaging tracers injected at very low concentrations are sufficient to provide high-quality images. However, this approach would prove to be problematic in the case of a dual-modality SPECT/MR imaging agent, since MR imaging requires much higher concentrations of injected contrast agent, in order to provide satisfactory images. Our MRI studies showed that a low-concentration nanomaterial injection (89μg/100μl/mouse, i.e., the injected dose of radiotracer for the gamma camera imaging) could not provide adequate contrast, while a high-concentration sample (1,000μg/100μl/mouse, Figure 8) was sufficient for contrast. For future applications, we will opt to work with nanoparticle samples having an adequately high concentration for satisfactory MR imaging, and to adjust the amount of radioisotope to provide an imaging tracer for simultaneous SPECT/MR imaging.

### Histopathology

**Figure 9:**
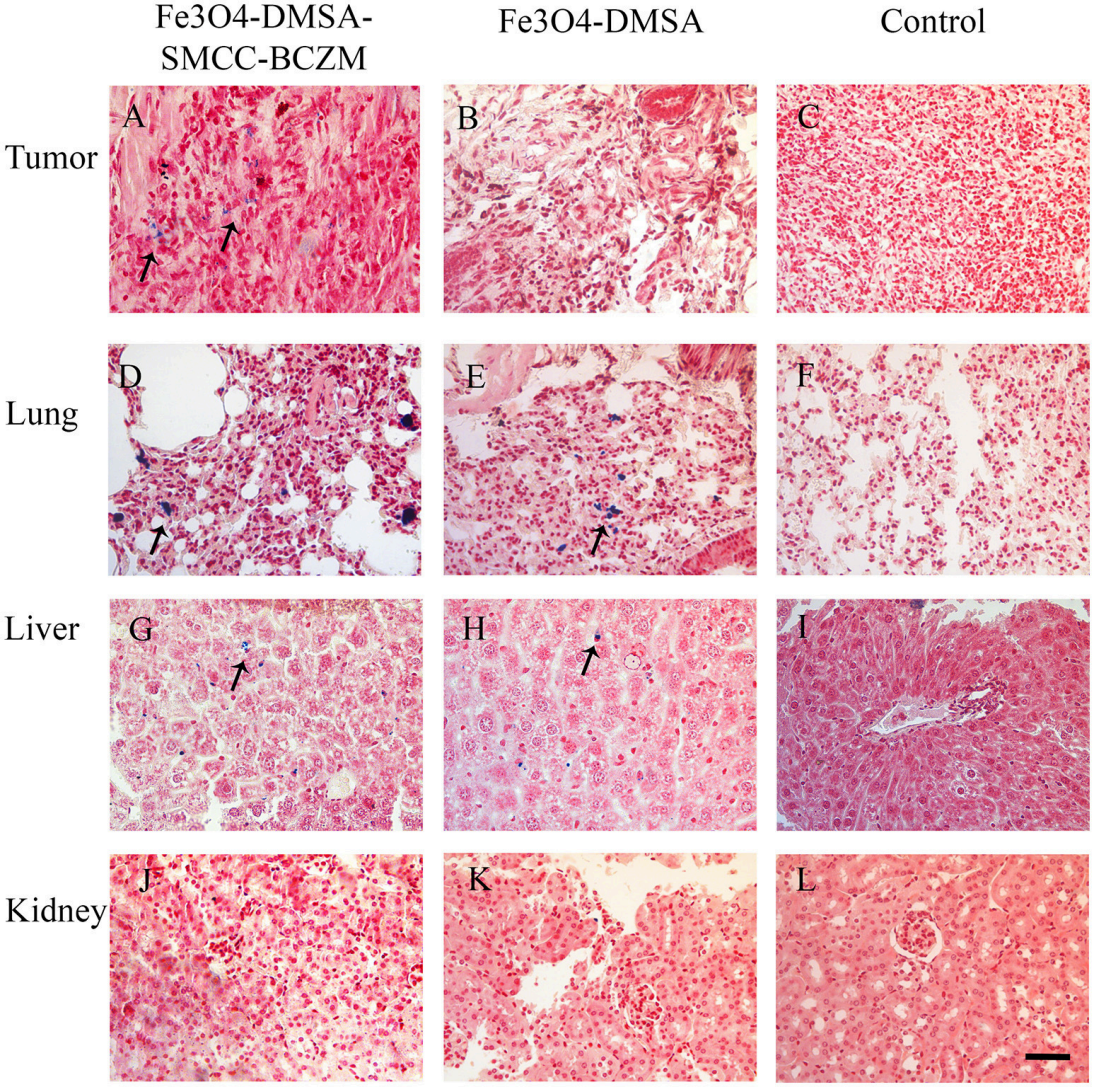
Prussian Blue staining of iron in tumor, liver, lung, and kidney sections of mice injected with Fe_3_O_4_-DMSA-SMCC-BCZM **(A,D,G,J)**, Fe_3_O_4_-DMSA **(B,E,H,K)** and saline **(C,F,I,L)**. The arrows indicate the accumulation of the iron nanoparticles. It is clearly evident that the functionalized nanoparticles Fe_3_O_4_-DMSA-SMCC-BCZM showed significant accumulation within the tumor. The scale bar is 20 μm.

Prussian blue staining of resected tumors from mice injected intravenously with non-functionalized Fe_3_O_4_-DMSA demonstrated little iron accumulation (Figure 9B), while Fe_3_O_4_-DMSA-SMCC-BCZM showed significant accumulation of iron within the tumor, suggesting improved tumor targeting for the antibody-functionalized nanoparticles (Figure 9A). Iron uptake was strong in the lungs of mice injected with both nanoconstructs (Figures 9D,E), while liver (Figures 9G,H) and kidneys (Figures 9J,K) also showed similar nanoparticle accumulation. Staining of tissues from mice injected with saline (control mice) showed no evidence of nanoparticle accumulation (Figures 9C,F,I,L). These findings are in line with our *ex vivo* and *in vivo* studies, and provide further proof of the targeting capabilities of Fe_3_O_4_-DMSA-SMCC-BCZM.

By performing this study, our intention was to show that the antibody-functionalized Fe_3_O_4_-DMSA-SMCC-BCZM exhibited targeted delivery to M165 tumors, while the non-functionalized nanoconstruct Fe_3_O_4_-DMSA did not. We believe that both our *ex vivo* and *in vivo imaging* experiments, as well as the performed histopathology study, have proven the concept of our hypothesis.

## Conclusions

In this proof-of-concept study, we have successfully prepared and tested a dual-modality nanoplatform functionalized with the monoclonal antibody BCZM. We have demonstrated that our nanoconstruct does not cause toxic effects in normal and cancer cells and has the ability to bind to the VEGF-165 isoform overexpressed on M165 tumor cells, when labeled with ^99m^Tc, forming stable constructs. *Ex vivo* biodistribution studies showed that the tumor-to-blood and the tumor-to-muscle ratios reached a maximum at 24 h p.i. (~7 and ~18 respectively), confirming high specificity of the antibody-functionalized tracer toward the overexpressed VEGF-165 isoform. Initial pilot *in vivo* images were in line with the *ex vivo* results, which were further proven by the histopathology study. The overall encouraging preliminary results we have obtained warrant further investigation into the dual-modality capabilities of the developed probes. However, further modifications may be required to improve the *in vivo* behavior of these nanoconstructs.

The primary idea behind the design and synthesis of these NPs was to provide a platform for the development of a dual-modality molecular imaging agent. We envision that once the targeted nanoconstruct is efficiently delivered to its target, it can be developed into a therapeutic tool, by exploiting its magnetic properties after application of an external magnetic field to heat tissue or activate drug release. The theranostic potential of these NPs could be further enhanced after radiolabeling with Rhenium-188 (^188^Re), a therapeutic isotope with chemical properties similar to ^99m^Tc.

## Author contributions

CT, DS, and PB designed the study and analyzed the data. All authors contributed to the experimental execution of the study, and to the writing process of the manuscript. All authors have read and approved of the final version of the manuscript.

### Conflict of interest statement

The authors declare that the research was conducted in the absence of any commercial or financial relationships that could be construed as a potential conflict of interest.
