# Supplementary material for: A Novel Metal-Based Imaging Probe for Targeted Dual-Modality SPECT/MR Imaging of Angiogenesis
Source: Front Chem. 2018 Jun 20;6:224. doi: 10.3389/fchem.2018.00224 (PMC6019489; doi:10.3389/fchem.2018.00224)
Supplement: Supplementary file 1 [file Data_Sheet_1.DOCX]

**Electronic Supplementary Material**

**A novel metal-based imaging probe for targeted**

**dual-modality SPECT/MR imaging of angiogenesis**

**Journal: Frontiers in Chemistry**

*C. Tsoukalas^1^, D. Psimadas^1^, G. A. Kastis^1,2^, V. Koutoulides^3^, A.L. Harris^4^,*

*M. Paravatou-Petsotas^1^, M. Karageorgou^1,5^, L.R. Furenlid^6,7^, L.A. Moulopoulos^3^,*

*D. Stamopoulos^5,8^, P. Bouziotis^1^*

^1^Radiochemical Studies Laboratory, Institute of Nuclear & Radiological Sciences & Technology, Energy & Safety, National Center for Scientific Research “Demokritos”, Athens, Greece

^2^Research Center of Mathematics, Academy of Athens, Athens, Greece

^3^First Department of Radiology, School of Medicine, National and Kapodistrian University of Athens, Athens, Greece

^4^Weatherall Institute of Molecular Medicine, University of Oxford, Oxford, U.K.;

^5^Department of Solid State Physics, NKUA, Athens, Greece

^6^Center for Gamma-Ray Imaging, Department of Medical Imaging, University of Arizona, Tucson, Arizona, USA

^7^College of Optical Sciences, University of Arizona, Tucson, Arizona, USA

^8^Institute of Nanoscience & Nanotechnology, National Center for Scientific Research “Demokritos”, Athens, Greece

***Corresponding author**: Dr. Penelope Bouziotis, E-mail: bouzioti@rrp.demokritos.gr; Phone: : +30 210 6503687, 0030 6973348788; Fax: +30 210 6545496


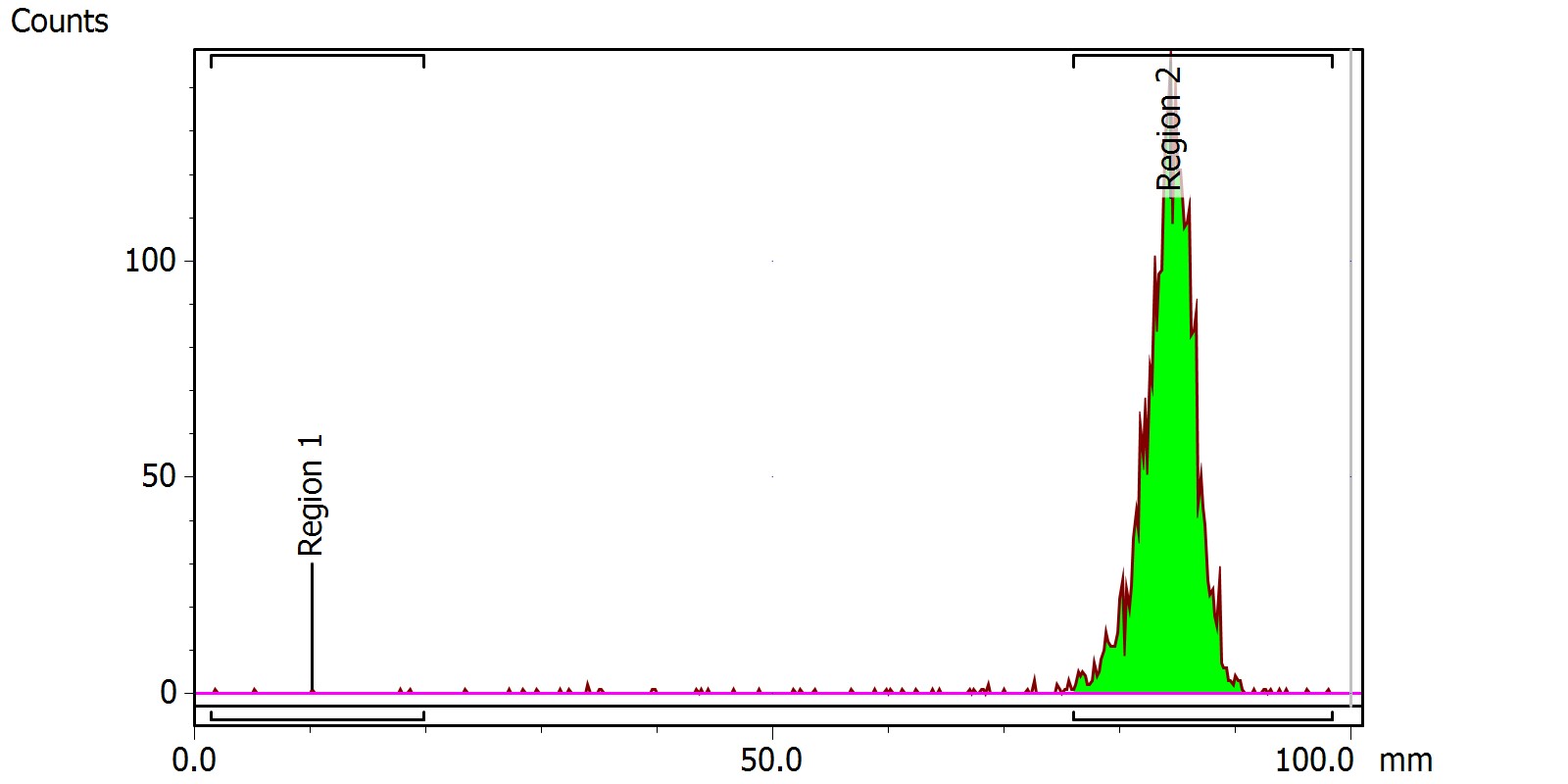


**A**


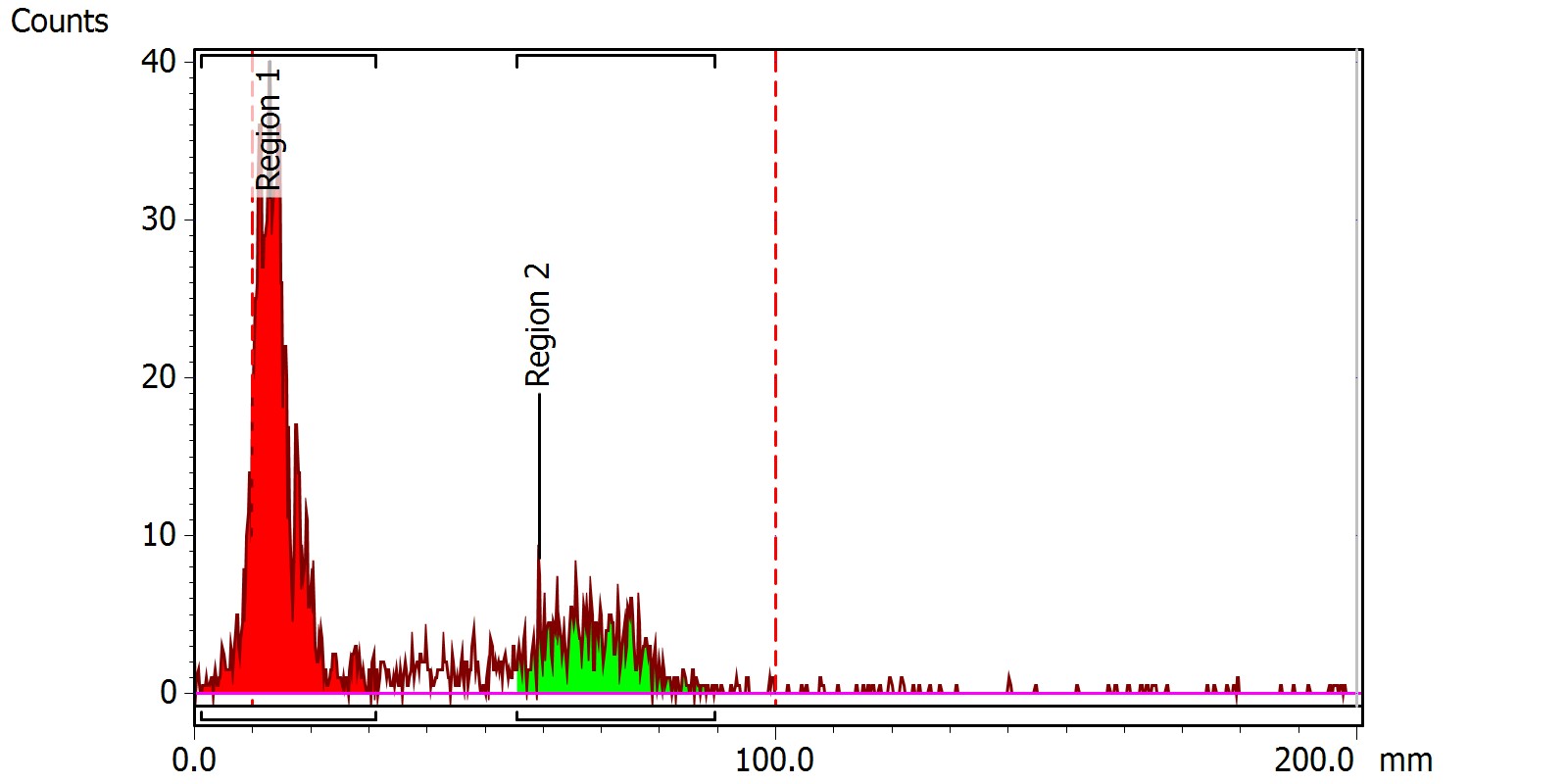


**B**


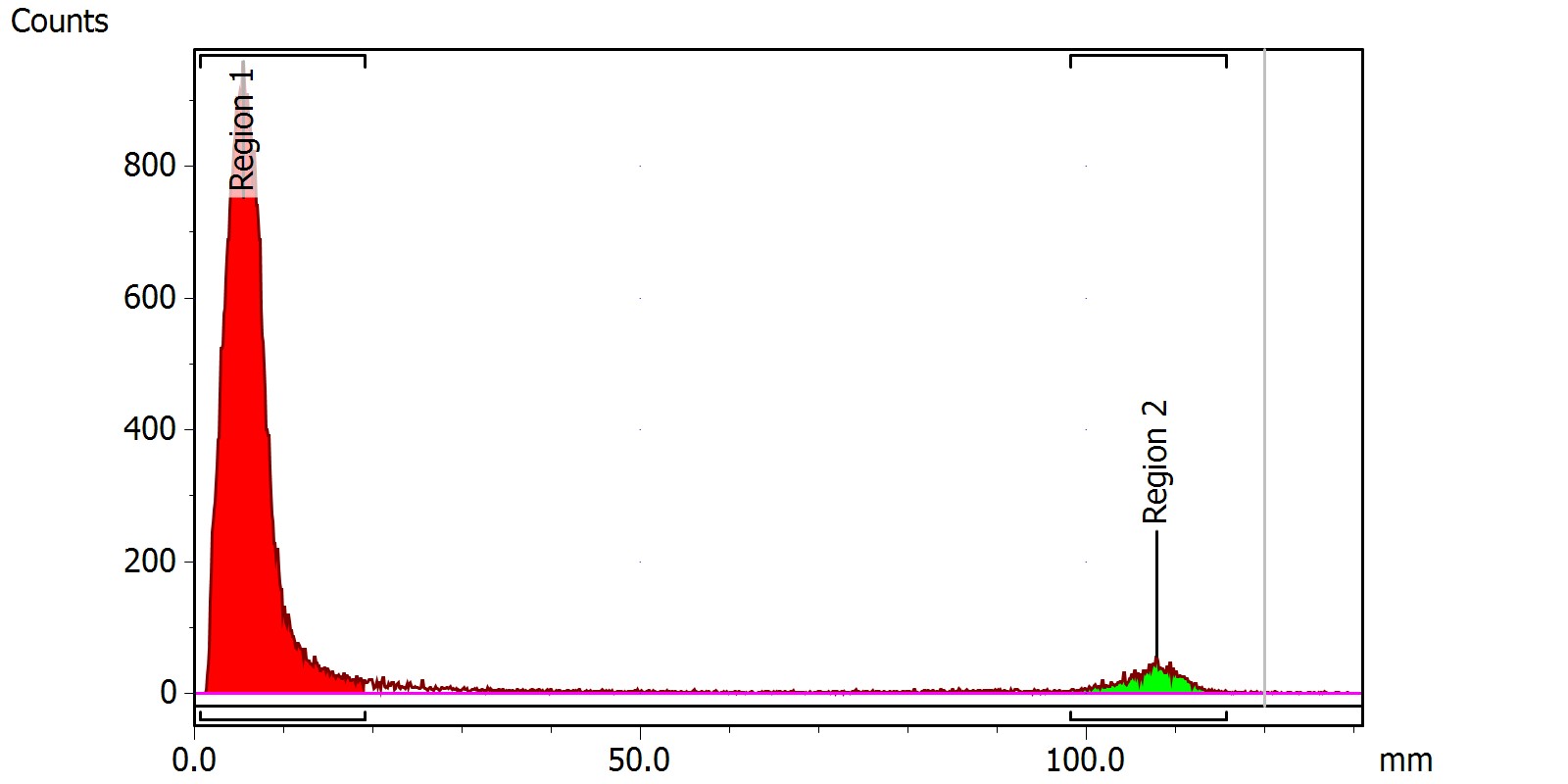


**C**

**Figure S1**: TLC evaluation of Fe_3_O_4_-DMSA radiolabeling with ^99m^Tc: Stationary phase: ITLC-SG; Mobile phase: MEK. A) Free ^99m^TcO_4_^-^ (control): Origin 0.16%, Front 99.84%; B) Fe_3_O_4_-DMSA-^99m^Tc (pre-purification): Origin: 72.62%, Front 27.38%; C) Fe_3_O_4_-DMSA-^99m^Tc (post-purification by magnetic retraction): Origin 94.38%, Front 5.62%.

**A**

17.06 min

9.58 min

11.52 min

**B**

**Figure S2**. HPLC radiochromatograms of: A) ^99m^Tc-gluconate; and B) Radiochemical purity of SMCC-BCZM-^99m^Tc. The peak at 11.52 represents SMCC-BCZM-^99m^Tc (96.08%), while the small peak can be attributed to the formation of antibody dimers (3.92%).


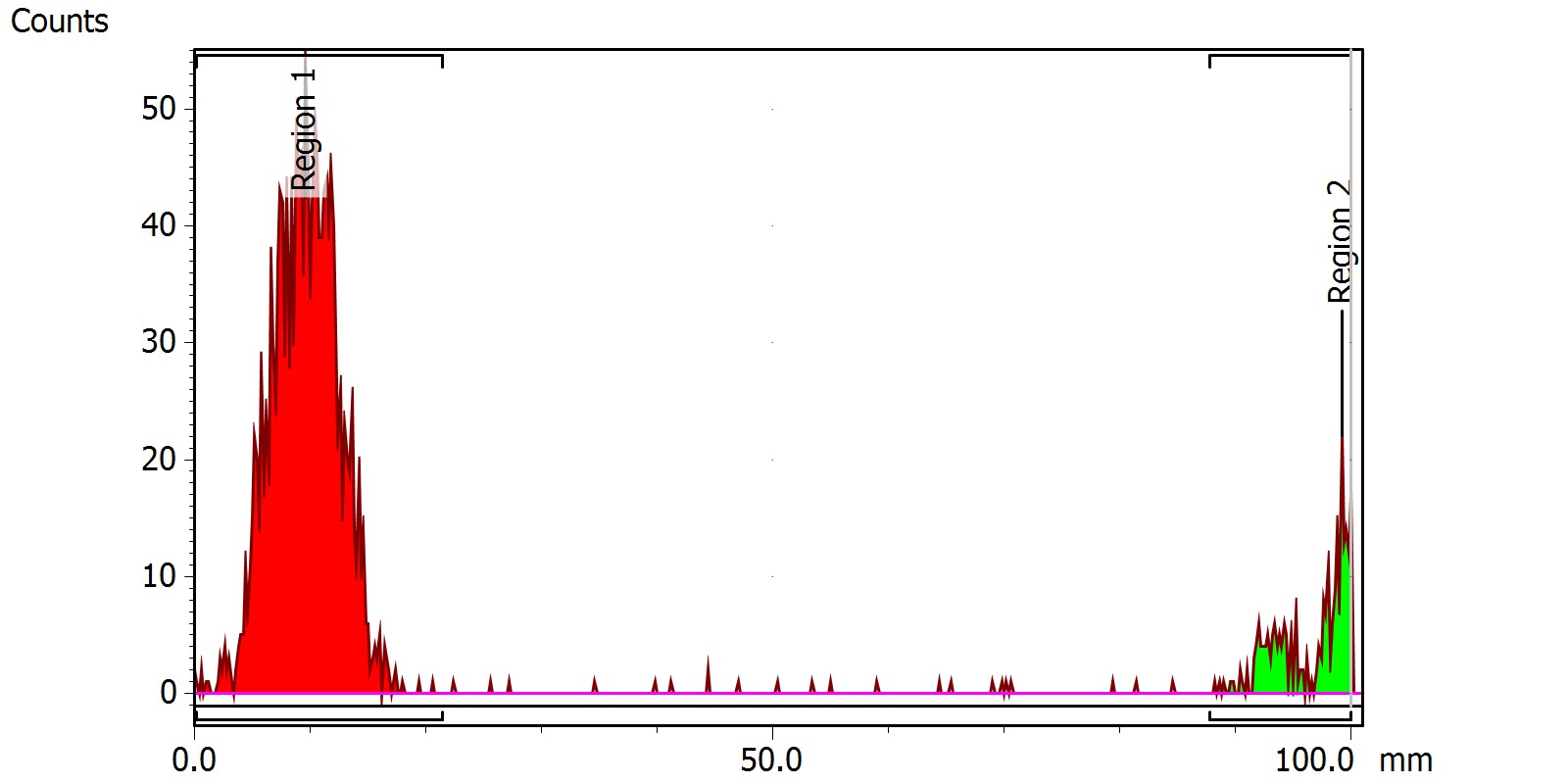


**A**


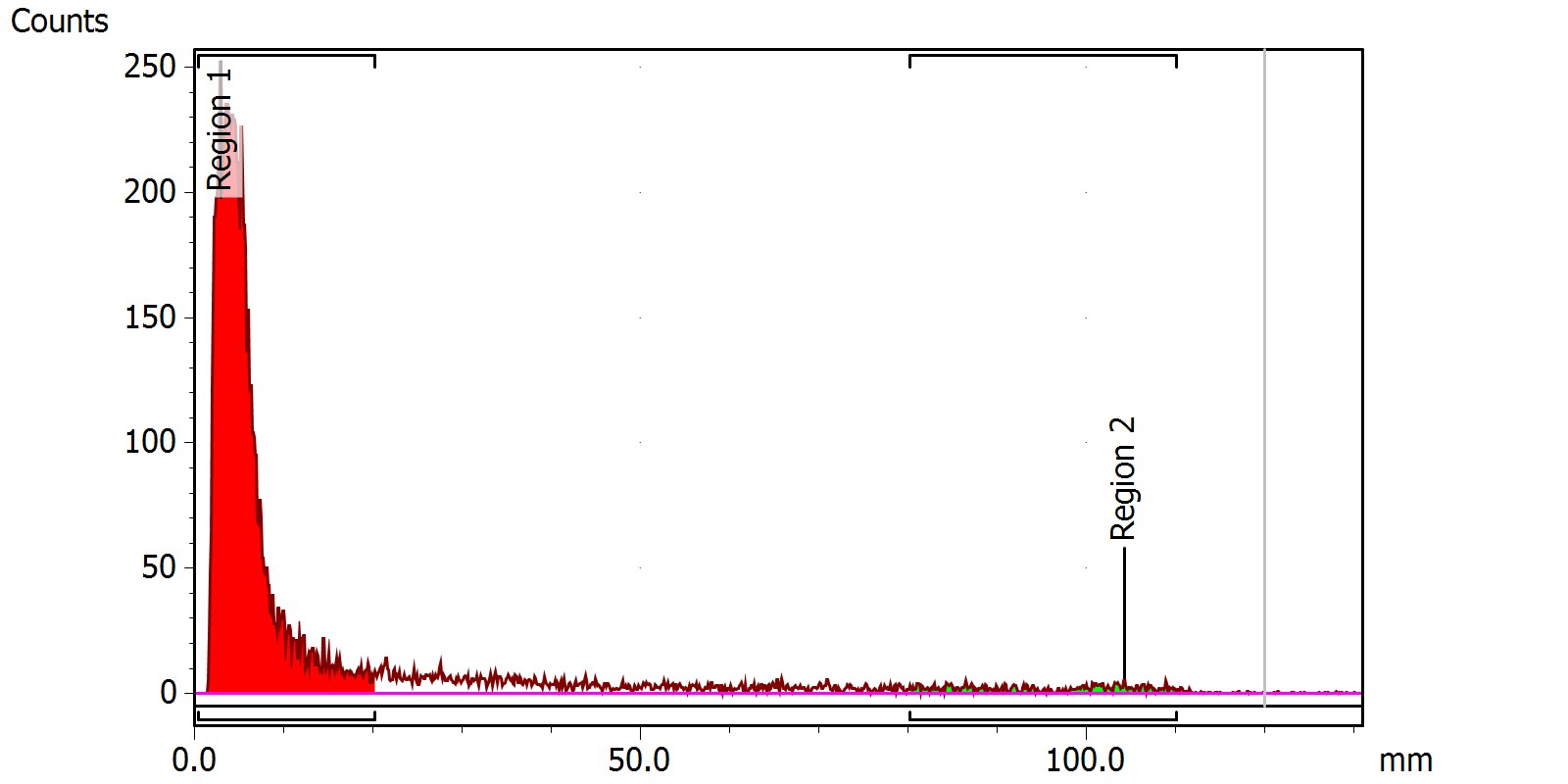


**B**

**Figure S3**: TLC evaluation of Fe_3_O_4_-DMSA radiolabeling with SMCC-BCZM-^99m^Tc: Stationary phase: ITLC-SG; Mobile phase: MEK. A) Fe_3_O_4_-DMSA-SMCC-BCZM-^99m^Tc (pre-purification): Origin 87.43%, Front 12.57%; B) Fe_3_O_4_-DMSA-SMCC-BCZM-^99m^Tc (post-purification by magnetic retraction): Origin 94.80%, Front: 5.20%.


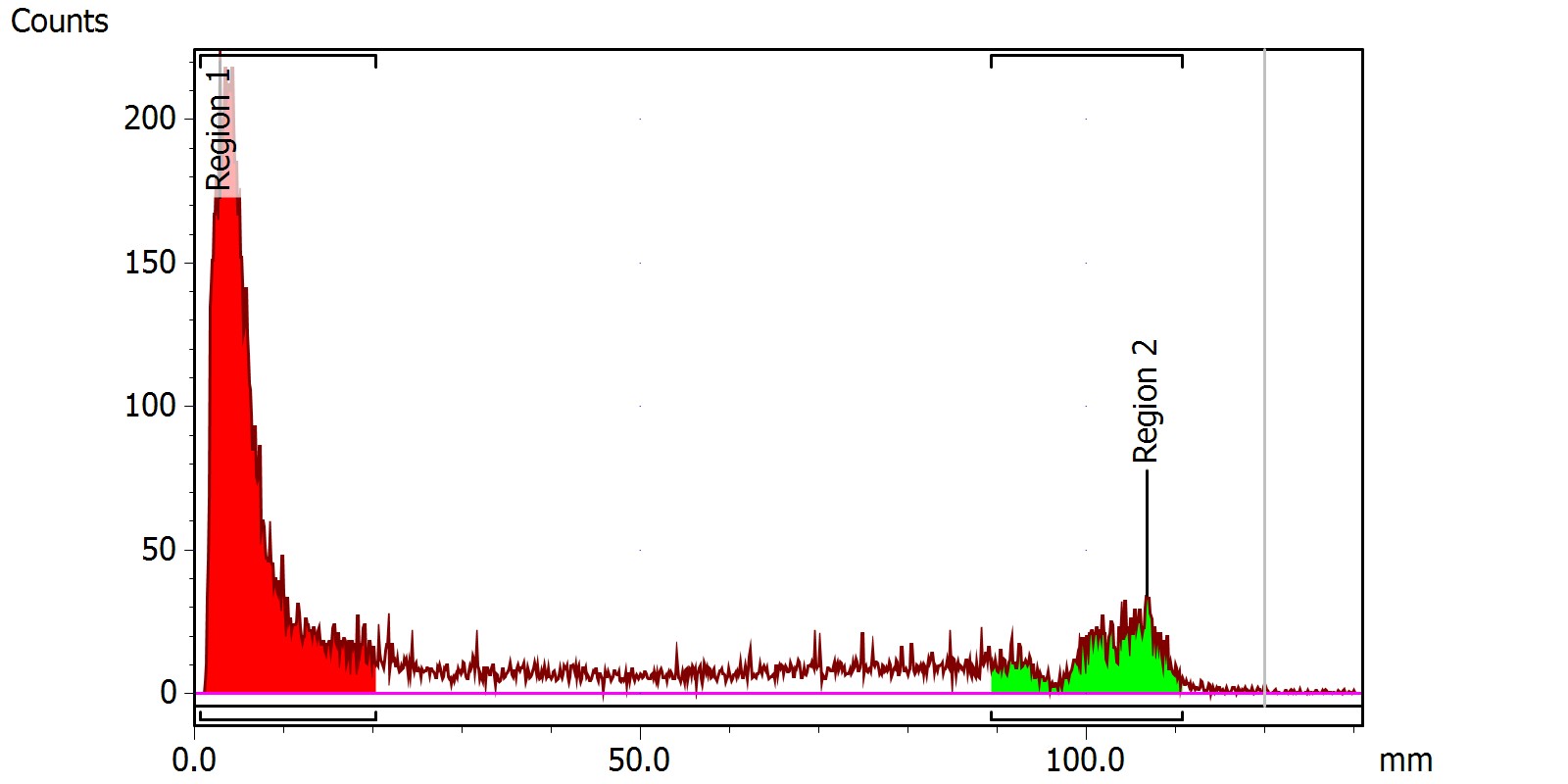


**Figure S4**. Stability in PBS at 24h post-incubation: Origin 80.71%, Front 19.29%


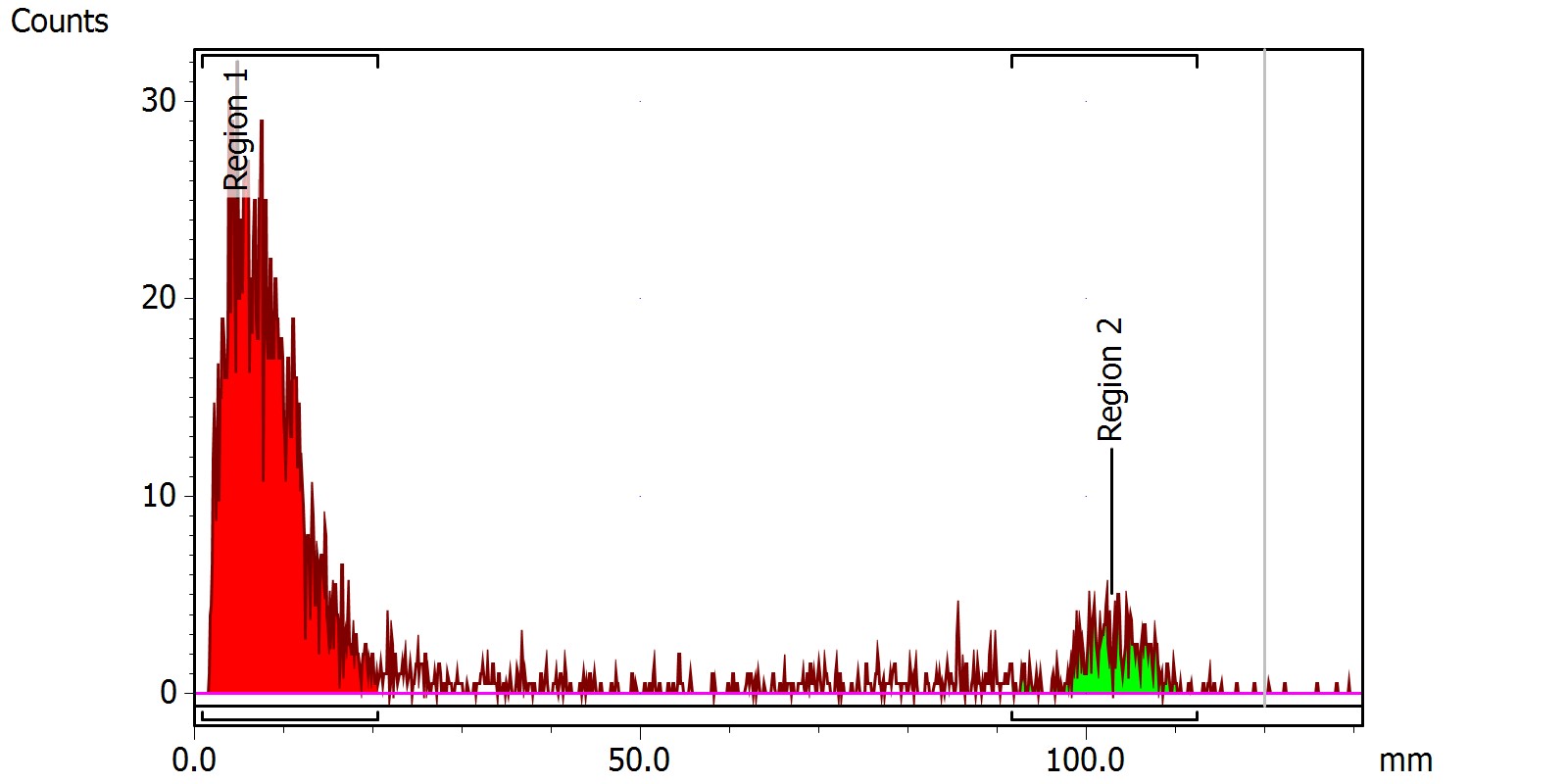


**Figure S5** Stability in serum at 24h post-incubation: Origin 90.25%, Front 9.75%

F

**Table S1.** Biodistribution of Fe_3_O_4_-DMSA-SMCC-BCZM-^99m^Tc and Fe_3_O_4_-DMSA-^99m^Tc in M165 tumor-bearing SCID mice at 2, 4 and 24 h post-injection.

|  | **2 h** | | | | | | **4 h** | | | | | | **24 h** | | | | | |
| --- | --- | --- | --- | --- | --- | --- | --- | --- | --- | --- | --- | --- | --- | --- | --- | --- | --- | --- |
|  | **Fe_3_O_4_-DMSA-SMCC-BCZM-^99m^Tc** | | | **Fe_3_O_4_-DMSA- ^99m^Tc** | | | **Fe_3_O_4_-DMSA-SMCC-BCZM-^99m^Tc** | | | **Fe_3_O_4_-DMSA- ^99m^Tc** | | | **Fe_3_O_4_-DMSA-SMCC-BCZM-^99m^Tc** | | | **Fe_3_O_4_-DMSA- ^99m^Tc** | | |
| **Blood** | 9.90 | ± | 2.07 | 3.21 | ± | 0.65 | 7.64 | ± | 0.76 | 2.96 | ± | 0.12 | 0.82 | ± | 0.08 | 1.54 | ± | 0.24 |
| **Heart** | 2.76 | ± | 0.33 | 2.47 | ± | 0.91 | 2.51 | ± | 0.50 | 2.99 | ± | 0.91 | 0.65 | ± | 0.16 | 0.89 | ± | 0.15 |
| **Liver** | 17.77 | ± | 3.61 | 23.60 | ± | 3.90 | 15.88 | ± | 2.09 | 26.84 | ± | 3.77 | 7.81 | ± | 1.25 | 17.33 | ± | 1.03 |
| **Spleen** | 16.88 | ± | 5.81 | 8.14 | ± | 2.49 | 13.00 | ± | 1.39 | 6.30 | ± | 3.10 | 5.38 | ± | 0.73 | 8.10 | ± | 2.88 |
| **Lung** | 29.04 | ± | 4.21 | 64.51 | ± | 8.44 | 24.26 | ± | 9.11 | 52.15 | ± | 0.08 | 9.04 | ± | 4.34 | 28.27 | ± | 1.45 |
| **Kidney** | 16.46 | ± | 2.79 | 8.59 | ± | 0.45 | 15.22 | ± | 5.37 | 7.83 | ± | 0.39 | 6.85 | ± | 0.13 | 7.48 | ± | 1.22 |
| **Stomach** | 2.87 | ± | 1.26 | 1.39 | ± | 0.40 | 2.91 | ± | 1.05 | 1.48 | ± | 0.30 | 1.50 | ± | 0.57 | 0.77 | ± | 0.11 |
| **Intestine** | 4.00 | ± | 0.75 | 1.07 | ± | 0.07 | 4.94 | ± | 1.17 | 1.24 | ± | 0.70 | 0.33 | ± | 0.05 | 0.95 | ± | 0.34 |
| **Pancreas** | 2.55 | ± | 0.38 | 0.70 | ± | 0.24 | 2.01 | ± | 0.37 | 0.52 | ± | 0.05 | 0.39 | ± | 0.02 | 0.58 | ± | 0.16 |
| **Muscle** | 1.43 | ± | 0.69 | 0.55 | ± | 0.05 | 0.99 | ± | 0.35 | 0.51 | ± | 0.09 | 0.35 | ± | 0.05 | 0.38 | ± | 0.19 |
| **Tumor** | 8.91 | ± | 1.88 | 2.06 | ± | 0.34 | 16.21 | ± | 2.56 | 1.92 | ± | 0.34 | 6.01 | ± | 1.69 | 1.88 | ± | 0.20 |

**Table S2.** Tumor/Blood and Tumor/Muscle ratios for Fe_3_O_4_-DMSA-SMCC-BCZM-^99m^Tc and Fe_3_O_4_-DMSA-^99m^Tc at 2, 4 and 24 h post-injection.

|  | **2 h** | | | | | | **4 h** | | | | | | **24 h** | | | | | |
| --- | --- | --- | --- | --- | --- | --- | --- | --- | --- | --- | --- | --- | --- | --- | --- | --- | --- | --- |
|  | **Fe_3_O_4_-DMSA-SMCC-BCZM-^99m^Tc** | | | **Fe_3_O_4_-DMSA- ^99m^Tc** | | | **Fe_3_O_4_-DMSA-SMCC-BCZM-^99m^Tc** | | | **Fe_3_O_4_-DMSA- ^99m^Tc** | | | **Fe_3_O_4_-DMSA-SMCC-BCZM-^99m^Tc** | | | **Fe_3_O_4_-DMSA- ^99m^Tc** | | |
| **Tumor/ Blood** | 0.90 | ± | 0.13 | 0.64 | ± | 0.22 | 2.12 | ± | 1.27 | 0.65 | ± | 0.74 | 7.35 | ± | 1.14 | 1.22 | ± | 0.24 |
|  |  |  |  |  |  |  |  |  |  |  |  |  |  |  |  |  |  |  |
| **Tumor/ Muscle** | 6.24 | ± | 0.84 | 3.74 | ± | 0.20 | 7.37 | ± | 1.57 | 3.75 | ± | 0.18 | 17.10 | ± | 1.16 | 5.02 | ± | 0.003 |


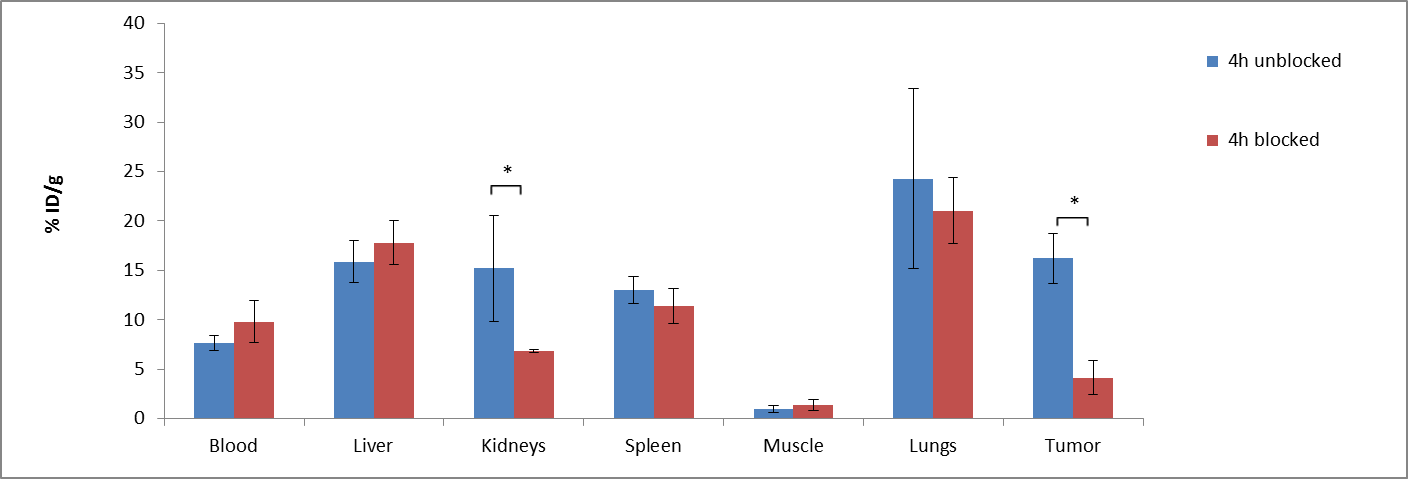


**Fig. S6**. Blocking studies of Fe_3_O_4_-DMSA-SMCC-BCZM-^99m^Tc in M165 tumor-bearing SCID mice at 4 h post-injection. Mean (%ID/g) ± SD (n = four animals); significant differences (P < 0.05) are denoted with an asterisk.

**Table S3.** Blocking studies of Fe_3_O_4_-DMSA-SMCC-BCZM-^99m^Tc and Fe_3_O_4_-DMSA-^99m^Tc in M165 tumor-bearing SCID mice at 4 h post-injection.

|  | **4 h** | | | | | |
| --- | --- | --- | --- | --- | --- | --- |
|  | **Fe_3_O_4_-DMSA-SMCC-BCZM-^99m^Tc** | | | **Fe_3_O_4_-DMSA- ^99m^Tc** | | |
| **Blood** | 7.64 | ± | 0.76 | 9.82 | ± | 2.08 |
| **Heart** | 2.51 | ± | 0.50 | 2.65 | ± | 0.16 |
| **Liver** | 15.88 | ± | 2.09 | 17.81 | ± | 2.25 |
| **Spleen** | 13.00 | ± | 1.39 | 11.38 | ± | 1.73 |
| **Lung** | 24.26 | ± | 9.11 | 21.04 | ± | 4.34 |
| **Kidney** | 15.22 | ± | 5.37 | 6.85 | ± | 0.13 |
| **Stomach** | 2.91 | ± | 1.05 | 1.50 | ± | 0.57 |
| **Intestine** | 4.94 | ± | 1.17 | 0.33 | ± | 0.05 |
| **Pancreas** | 2.01 | ± | 0.37 | 0.39 | ± | 0.02 |
| **Muscle** | 0.99 | ± | 0.35 | 0.35 | ± | 0.05 |
| **Tumor** | 16.21 | ± | 2.56 | 4.11 | ± | 1.72 |
